# Preclinical evaluation of a TEX101 protein ELISA test for the differential diagnosis of male infertility

**DOI:** 10.1186/s12916-017-0817-5

**Published:** 2017-03-23

**Authors:** Dimitrios Korbakis, Christina Schiza, Davor Brinc, Antoninus Soosaipillai, Theano D. Karakosta, Christine Légaré, Robert Sullivan, Brendan Mullen, Keith Jarvi, Eleftherios P. Diamandis, Andrei P. Drabovich

**Affiliations:** 1 Department of Laboratory Medicine and Pathobiology, University of Toronto, Toronto, ON M5T 3L9 Canada; 2 Lunenfeld-Tanenbaum Research Institute, Mount Sinai Hospital, Toronto, ON M5T 3L9 Canada; 3 Department of Pathology and Laboratory Medicine, Mount Sinai Hospital, Toronto, ON M5T 3L9 Canada; 4 Department of Clinical Biochemistry, University Health Network, Toronto, Canada; 5 Centre de Recherche du Centre Hospitalier Universitaire (CHU) de Québec, Département d’Obstétrique, Gynécologie et Reproduction, Faculté de Medicine, Université Laval, Québec, Canada; 6 Division of Urology, Department of Surgery, Mount Sinai Hospital, Toronto, Canada

**Keywords:** Male infertility, Azoospermia, Oligospermia, TEX101, Testis-expressed sequence 101 protein, Mass spectrometry, Immunoassay, Seminal plasma, Seminal microvesicles

## Abstract

**Background:**

TEX101 is a cell membrane protein exclusively expressed by testicular germ cells and shed into seminal plasma. We previously verified human TEX101 as a biomarker for the differential diagnosis of azoospermia, and developed a first-of-its-kind TEX101 ELISA. To demonstrate the clinical utility of TEX101, in this work we aimed at evaluating ELISA performance in a large population of fertile, subfertile, and infertile men.

**Methods:**

Mass spectrometry, size-exclusion chromatography, ultracentrifugation, and immunohistochemistry were used to characterize TEX101 protein as an analyte in seminal plasma. Using the optimized protocol for seminal plasma pretreatment, TEX101 was measured by ELISA in 805 seminal plasma samples.

**Results:**

We demonstrated that TEX101 was present in seminal plasma mostly in a free soluble form and that its small fraction was associated with seminal microvesicles. TEX101 median values were estimated in healthy, fertile pre-vasectomy men (5436 ng/mL, *N* = 64) and in patients with unexplained infertility (4967 ng/mL, *N* = 277), oligospermia (450 ng/mL, *N* = 270), and azoospermia (0.5 ng/mL, *N* = 137). Fertile post-vasectomy men (*N* = 57) and patients with Sertoli cell-only syndrome (*N* = 13) and obstructive azoospermia (*N* = 36) had undetectable levels of TEX101 (≤0.5 ng/mL). A cut-off value of 0.9 ng/mL provided 100% sensitivity at 100% specificity for distinguishing pre- and post-vasectomy men. The combination of a concentration of TEX101 > 0.9 ng/mL and epididymis-specific protein ECM1 > 2.3 μg/mL provided 81% sensitivity at 100% specificity for differentiating between non-obstructive and obstructive azoospermia, thus eliminating the majority of diagnostic testicular biopsies. In addition, a cut-off value of ≥0.6 ng/mL provided 73% sensitivity at 64% specificity for predicting sperm or spermatid retrieval in patients with non-obstructive azoospermia.

**Conclusions:**

We demonstrated the clinical utility of TEX101 ELISA as a test to evaluate vasectomy success, to stratify azoospermia forms, and to better select patients for sperm retrieval.

**Electronic supplementary material:**

The online version of this article (doi:10.1186/s12916-017-0817-5) contains supplementary material, which is available to authorized users.

## Background

Infertility is a common medical condition with an estimated prevalence of nearly 15% in the general population [1]. The disorder affects both men and women, while the male factor, exclusively or combined with female abnormalities, contributes to approximately 50% of all cases. The clinical categories of male infertility range from lowered production of sperm, or oligospermia, to severe cases of azoospermia with non-measurable levels of sperm in semen [2]. Azoospermia is diagnosed in nearly 2% of the general population and has two major forms, non-obstructive (NOA) and obstructive azoospermia (OA). Based on histological evaluation of testicular tissue, the NOA subtype is further classified into hypospermatogenesis (HS), maturation arrest (MA), and Sertoli cell-only syndrome (SCO) [3]. OA results from physical obstruction in the male reproductive tract due to congenital or acquired defects in the epididymis or vas deferens [4]. A diagnostic testicular biopsy is used to evaluate the rate of spermatogenesis and the presence of sperm in the testis, and it remains the standard tool for differential diagnosis of azoospermia [5]. However, it is an invasive surgical procedure with potential complications. Thus, there is an urgent need for alternative, non-invasive approaches for differential diagnosis of male infertility and further classification of its subtypes.

Seminal plasma (SP) is enriched with testis-derived proteins, mRNA, and metabolites. It has been proposed as a suitable clinical sample for the non-invasive diagnosis of a wide range of male reproductive system disorders [6–8]. SP contains up to 3200 proteins secreted by testes, epididymis, prostate, seminal vesicles, and Cowper’s glands [9, 10]. Numerous proteins found in SP are directly involved in the production and maturation of sperm or in the interaction with the zona pellucida and fusion with oocytes [11]. Because testis-specific biomarkers are not found in blood serum due to stringent blood–testis and blood–epididymis barriers, semen and SP remain the only viable fluids for the non-invasive diagnosis of male infertility [12, 13].

We previously proposed a simple two-biomarker algorithm for the non-invasive differential diagnosis of azoospermia [14]. Two proteins, the testis-specific protein TEX101 and the epididymis-expressed protein ECM1 were verified and validated as biomarkers for the differential diagnosis of NOA versus OA [9, 11]. In addition, TEX101 levels in SP facilitated further classification of NOA subtypes into HS, MA, and SCO [14]. Mass spectrometry-based selected reaction monitoring (SRM) and immuno-SRM assays were initially used to measure TEX101 in SP, but translation of those assays into routine clinic practice required the higher throughput, better sensitivity, and greater simplicity of ELISA [15]. Recently, we generated monoclonal antibodies, developed TEX101 ELISA, and optimized SP handling and pretreatment protocols in order to increase assay sensitivity [16].

**Fig. 1:**
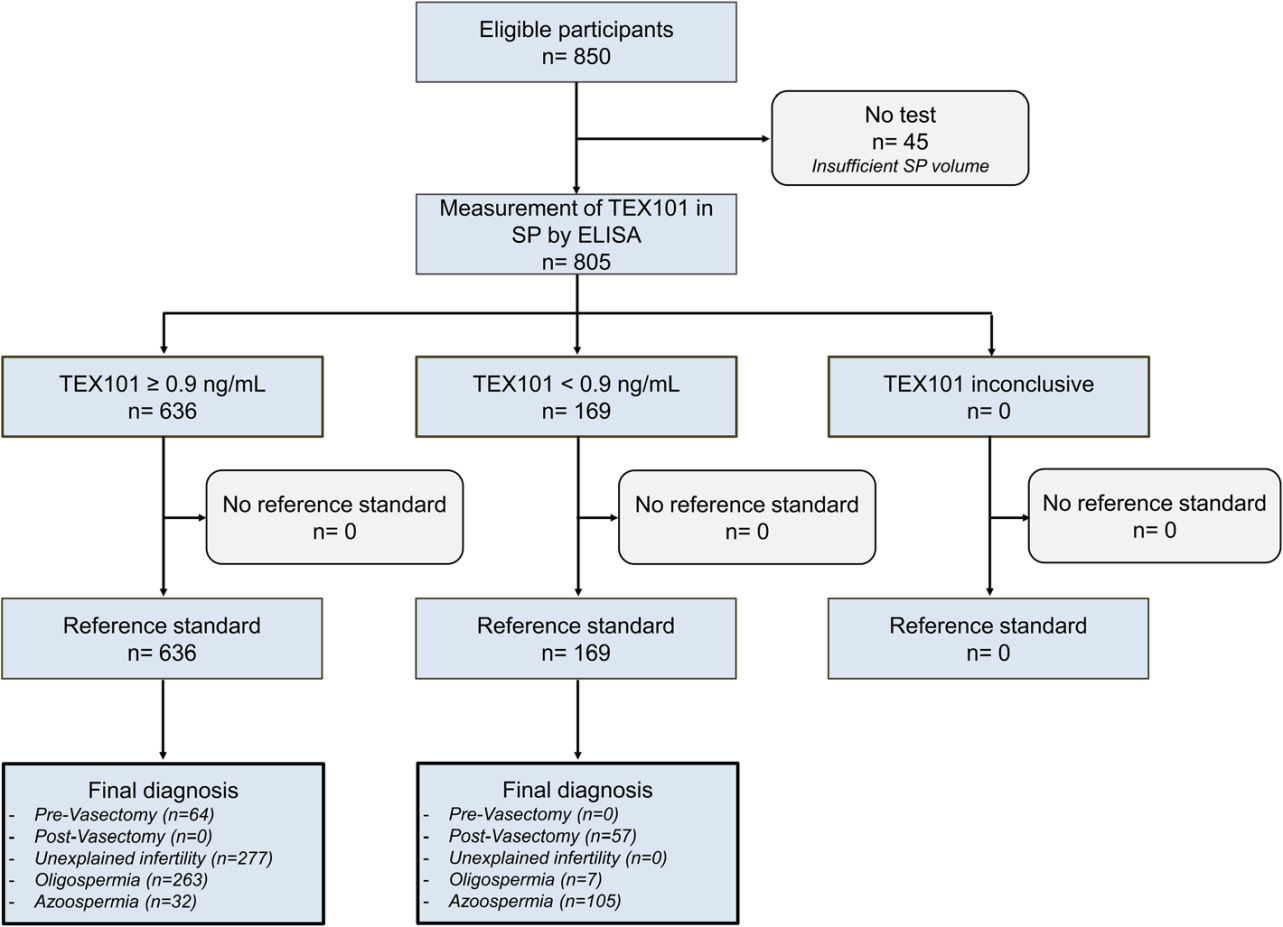
Flowchart of the study. *SP* seminal plasma

Previously, we proposed the clinical utility of TEX101 as a biomarker based on its measurements in SP by mass spectrometry [14]. In this work, we focused on the full characterization of TEX101 as an analyte in SP; the preclinical evaluation of the performance of TEX101 ELISA in a large cohort of fertile, subfertile, and infertile men; and the validation of TEX101 as a prognostic biomarker of male infertility and a predictive biomarker of sperm retrieval in NOA patients. Our objective was also to evaluate TEX101 ELISA as a test for the differential diagnosis of the most common clinical conditions of male infertility (unexplained infertility, oligospermia, and azoospermia) and to propose simple decision trees for use in the clinic. We hypothesized that TEX101 levels in SP would vary in patients with different categories of male infertility. We would suggest that the TEX101 test is offered after the standard protocols for the initial evaluation of infertility (semen analysis and measurement of motility, morphology, and levels of reproductive hormones) in patients admitted to urology clinics, but prior to diagnostic testicular biopsies. The presented study was based on retrospectively collected SP samples (Fig. 1) and followed the Standards for Reporting Diagnostic Accuracy Studies (STARD 2015) statement [17], to demonstrate the performance of our TEX101 ELISA, and to propose three distinct clinical utilities of our test.

## Methods

### Experimental design and statistical rationale

Based on our previous measurements of TEX101 by ELISA [16], the minimal sample size required to validate TEX101 performance in pre- and post-vasectomy groups was 18 samples (two-tailed Mann–Whitney U test, α = 0.05, 80% power, allocation ratio of 1). Even though the minimal sample size was small, we decided to measure TEX101 in all SP samples available in our biobank in order to establish more accurate clinical cut-off values.

### Patients with inclusion and exclusion criteria

Healthy, fertile men pre- and post-vasectomy, and men referred to Mount Sinai Hospital for clinical infertility evaluation were included in the study. Initial patient evaluation included computer-assisted semen analysis and measurement of reproductive hormones (testosterone, estradiol, follicle-stimulating hormone, luteinizing hormone, and prolactin). Sperm concentration, ejaculate volume, motility, and morphology were graded based on World Health Organization 5th edition criteria [18]. Azoospermia was defined as no sperm found on the initial semen analysis, and oligospermia included men with spermatozoa present at concentrations <15 million/mL. Unexplained infertility was defined as the inability to conceive after 1 year of regular unprotected intercourse, spermatozoa concentration >15 million/mL, and normal hormonal parameters. Note that we did not have female factor data for this group. There are a number of female infertility factors, such as tubal obstruction, endometriosis, polycystic ovary syndrome, among many others, that make female factor infertility a determinant or co-determinant. Thus, some patients in this group may in fact have been healthy, fertile men. Clinical reference standards for vasectomy were sperm counting, while clinical reference standards for OA, NOA and sperm retrieval were sperm counting, diagnostic testicular biopsies, and testicular sperm extraction (TESE). Clinical cut-off values were reported based on the randomized blind measurements of TEX101 in 805 retrospectively collected SP samples using ELISA with the sodium deoxycholate (DOC)-based protocol. The analysis did not include 45 patients for whom SP samples were fully consumed in the preliminary experiments.

### Seminal plasma samples

Following collection, semen was left to liquefy at room temperature (RT) for 1 h, then aliquoted and centrifuged three times at 13,000 × *g* for 15 min. SP was separated from cells and cellular debris and stored at −80 °C.

### TEX101 ELISA measurements

The 96-well ELISA plates were coated with 500 ng/well of mouse monoclonal anti-TEX101 antibody 23ED616.8 in 50 mM Tris-HCl buffer at pH 7.8. Plates were washed twice with the washing buffer (0.05% Tween20 in 20 mM Tris-HCl and 150 mM NaCl at pH 7.4). Assay calibrators were prepared as previously described [16]. Briefly, several dozen SP samples from fertile pre-vasectomy men were pooled, and endogenous TEX101 concentration (4.7 ± 1.5 μg/mL) was measured by SRM. Multiple 20 μL aliquots of the pool were stored at −20 °C. For ELISA measurements, calibrators and patient samples were thawed and mixed (1:1) with either Reagent mixture #1 (6 M guanidinium chloride [GndCl] at pH 12; 1 h incubation at RT) or Reagent mixture #2 (4% DOC in water; incubation for 1 h at 63 °C). Following treatment, calibration samples were diluted 100-fold with the assay diluent (60 g/L bovine serum albumin [BSA], 25 mL/L normal mouse serum, 100 mL/L normal goat serum, and 10 g/L bovine IgG in 50 mM Tris-HCl at pH 7.8). Subsequently, serial dilutions of the treated calibrator (0.5–50 ng/mL, 100 μL/well) were prepared with 4-fold dilution steps. Similarly, patient SP samples (20 μL) were treated with Reagent mixture #1 or 2 (1:1), further diluted 10-, 100-, 1000-, and 10,000-fold with the assay diluent, and added on ELISA plates (100 μL/well). Following 2 h of incubation with gentle shaking, plates were washed twice with the washing buffer. A biotinylated mouse monoclonal anti-TEX101 antibody 23ED660.7 in the assay diluent (250 ng in 100 μL per well) was added and incubated for 1 h. Plates were then washed six times, and streptavidin-conjugated alkaline phosphatase was added for 15 min with gentle shaking. After the final wash (six times), diflunisal phosphate solution was prepared in the substrate buffer (0.1 M NaCl, 1 mM MgCl_2_ in 0.1 M Tris at pH 9.1), added to the plate (100 μL per well), and incubated for 10 min at RT with gentle shaking. Finally, the developing solution (1 M Tris-HCl, 0.4 M NaOH, 2 mM TbCl_3_, and 3 mM EDTA) was added and mixed for 1 min. Time-resolved fluorescence was measured with the Wallac EnVision 2103 Multilabel Reader (Perkin Elmer), as previously described [19].

### Measurement of TEX101 isoforms in spermatozoa and seminal plasma by selected reaction monitoring

SP and spermatozoa of patients with a normal sperm count (median 26 million/mL; *N =* 17) were prepared. Spermatozoa were lysed by RapiGest SF Surfactant (Waters Corp., Milford, MA, USA). The total protein was measured by bicinchoninic acid (BCA) assay, and 5 and 10 μg of total protein of spermatozoa lysate and SP, respectively, were mixed with 50 mM ammonium bicarbonate. SRM assays were developed using previously described selectivity and reproducibility criteria [20–22]. Two hundred fmoles of the heavy isotope-labeled TEX101 peptide AGTETAILATK (present in both membrane/secreted isoform Q9BY14-1 and intracellular isoform Q9BY14-2) and 500 fmoles of heavy isotope-labeled peptide QIQTSSSQTSPEEAMGTPR (present exclusively in the intracellular isoform Q9BY14-2) were spiked before trypsin digestion. Heavy isotope-labeled peptides (SpikeTides^TM^_TQL, JPT Peptide Technologies GmbH, Berlin, Germany) included trypsin-cleavable quantifying JPT tags (serine-alanine-[3-nitro]tyrosine-glycine). Five millimoles of dithiothreitol (DTT) (Sigma-Aldrich, St. Louis, MO, USA) and 0.05% RapiGest SF Surfactant were added, and samples were incubated at 60 °C for 30 min. Following that, samples were alkylated in the dark with 10 mM iodoacetamide (Sigma-Aldrich) for 40 min and digested overnight at 37 °C with proteomics-grade porcine trypsin (Sigma-Aldrich, #T6567). Trypsin inactivation and cleavage of RapiGest SF was achieved with the addition of trifluoroacetic acid (1% final). Additionally, 5 mM of *L*-methionine (Sigma-Aldrich) was added to each digest, in order to limit the oxidation of methionine residues during sample preparation or storage. Finally, digests were loaded on C18 OMIX tips (Varian Inc., Lake Forest, CA, USA), and bound peptides were eluted in 3 μL of 65% acetonitrile in water with 0.1% formic acid. Water with 0.1% formic acid was added (60 μL final volume), and samples were transferred to the 96-well microplate (Axygen, Union City, CA, USA).

Using a 96-well microplate autosampler, 18 μL of each sample were loaded onto a 3 cm trap column (inner diameter 150 μm; New Objective, Woburn, MA, USA) packed in-house with 5 μm Pursuit C18 (Varian). An increasing concentration of Buffer B (0.1% formic acid in acetonitrile) was used to elute the peptides from the trap column onto a resolving analytical 5-cm PicoTip emitter column (inner diameter 75 μm, 8 μm tip; New Objective) packed in-house with 3 μm Pursuit C18 (Varian). The EASY-nLC system (Proxeon Biosystems) was coupled online to a TSQ Quantiva^TM^ triple quadrupole mass spectrometer (Thermo Scientific, San Jose, CA, USA) with a nanoelectrospray ionization source. The SRM parameters were as follows: positive polarity, declustering and entrance potentials of 150 and 10 V, respectively; ion transfer tube temperature 300 °C; optimized collision energy values; scan time 40 ms; 0.4 and 0.7 Da full width at half maximum resolution settings for the first and third quadrupoles, respectively; and 1.5 mTorr argon pressure in the second quadrupole. TEX101 peptides were monitored in a non-scheduled SRM mode during a 30 min LC gradient (Additional file 1: Table S1). The relative abundance of TEX101 in each sample was estimated as a ratio of the endogenous peptide to the spiked heavy isotope-labeled standards. Raw files for each sample were recorded and analyzed with Skyline software (v3.1.0.7382, MacCoss Lab Software, Seattle, WA, USA).

### Immunohistochemistry of TEX101 in testicular tissues

In-house-generated mouse monoclonal anti-TEX101 antibodies 23-ED-660, 23-ED-11, and 23-ED-228 were used to stain testicular tissue samples fixed with 10% buffered formalin. Samples were incubated with the antibody solutions for 1 h at RT. Multiple dilutions (400- to 5000-fold) were tested. Heat-induced epitope retrieval was performed in citrate buffer at pH 6.0. A Vectastain Elite ABC Kit (Vector Laboratories Inc., Burlingame, CA, USA), 3,3'-diaminobenzidine substrate (Sigma-Aldrich), and a LabVision 720 autostainer (Thermo Fisher Scientific Inc.) were used for detection.

### Size-exclusion chromatography

SP samples from pre-vasectomy men were centrifuged at 4000 × *g* for 20 min and pooled (total protein 39 mg/mL). A vesicle-free pool (total protein 22 mg/mL) was obtained by ultracentrifugation at 120,000 × *g*. Five hundred micrograms of total protein from both pools were diluted to 500 μl with the running buffer (0.1 M NaH_2_PO_4_-Na_2_HPO_4_ and 0.15 M NaCl at pH 7.0) and loaded on a TSKgel G3000SW size exclusion column (Tosoh Bioscience LLC, King of Prussia, PA, USA). Pools were run at 1 mL/min for 35 min, and fractions were collected every 0.5 min from 8 to 27 min. The presence of TEX101 in each fraction was measured by ELISA with DOC-based treatment.

### Isolation of seminal microvesicles from seminal plasma samples

Pre- and post-vasectomy SP samples, as well as four samples obtained from the group of infertile individuals with moderate-to-high sperm count, were individually pooled. Seminal microvesicles (SMVs) were isolated with a method adapted from Fabiani et al. [23]. Semen samples were centrifuged at 4000 × *g* for 20 min at 4 °C to remove cells and cell debris. The remaining supernatants were diluted 1:2 in a solution containing 30 mM Tris and 130 mM NaCl at pH 7.5 and centrifuged one more time at 4000 × *g* for 20 min at 4 °C. Following that, supernatants were ultracentrifuged at 120,000 × *g* for 2 h at 4 °C, pellets were re-suspended in 30 mM Tris and 130 mM NaCl at pH 7.5, and finally subjected to size-exclusion chromatography on a Sephacryl S-500 HR (15 × 85 mm; Pharmacia Canada Ltd, Dorval, QC, Canada) for membranous vesicle purification. The eluate was collected into 18 fractions (0.5 mL). The SMV-positive fractions were detected by elevated absorbance at 280 nm. Fractions 8–15 were pooled (4 mL) and ultracentrifuged at 120,000 × *g* for 2 h at 4 °C to pellet the SMVs. The pellets were resuspended in phosphate-buffered saline (PBS) at pH 7.4 and stored with matched vesicle-free SP samples at −80 °C. The amount of total protein was assessed by BCA assay (Pierce Biotechnology, Rockford, IL, USA).

### TEX101 measurement in pooled seminal plasma samples, seminal microvesicles, and vesicle-free seminal plasma by ELISA

SMVs, vesicle-free SP, and the original pooled pre- or post-vasectomy or infertile SP samples were mixed with corresponding reagent mixes (1:1) and incubated for 1 h either at RT or at 63 °C. Mixtures were further diluted with ELISA diluents before loading on the plate. Analysis was accomplished as described above.

### TEX101 measurement in pooled seminal plasma samples, seminal microvesicles, and vesicle-free seminal plasma by selected reaction monitoring

TEX101 concentrations in the original SP pools, SMVs, and vesicle-free fractions were calculated using the SRM assay described above. Ten micrograms of total protein from each sample was subjected to trypsin digestion. Heavy isotope-labeled peptide with a trypsin-cleavable tag AGTETAILATK*-JPTtag was used as an internal standard for absolute quantification of TEX101 protein.

### TEX101 measurement in seminal plasma by immunocapture-selected reaction monitoring

Immunocapture-SRM assay was used to investigate the effect of SP pretreatment on antibody–antigen interactions. Initially, a pool of SP was prepared and subjected to various types of treatment prior to analysis. Treatment options included: (i) mixing (1:1) with 6 M GndCl (pH 12) and incubation at RT for 1 h, (ii) mixing (1:1) with 4% DOC and incubation at RT for 1 h, (iii) mixing (1:1) with 6 M GndCl (pH 12) and incubation at 63 °C for 1 h, (iv) mixing (1:1) with 4% DOC and incubation at 63 °C for 1 h, and (v) incubation at 63 °C for 1 h. Non-treated SP was also included in the analysis. According to the established protocol [16], white 96-well microtiter plates were coated with 500 ng per well of purified mouse immunoglobulins in 50 mM Tris-HCl at pH 7.8. Antibodies used for coating included a commercial mouse polyclonal anti-TEX101 antibody (ab69522; Abcam, Cambridge, MA, USA), an in-house-generated mouse monoclonal anti-TEX101 antibody (23ED616.8), and a mouse IgG (Equitech-Bio, Inc., Cat. #M60) as an isotype control. Following overnight incubation at RT, plates were washed twice with PBS. Three dilutions (×10, ×100, ×1000) of pretreated SP samples in 6% BSA in PBS were loaded onto the plate (100 μL per well) and incubated for 2 h at RT with gentle shaking. Following that, plates were washed three times with PBS and three times with 50 mM ammonium bicarbonate. A mix containing 50 mM ammonium bicarbonate, 5 mM DTT, and 100 fmoles of heavy isotope-labeled TEX101 peptide AGTETAILATK*-JPTtag was added to each well and incubated for 30 min at RT. Subsequently, 10 mM iodoacetamide was added and samples were kept for 40 min in the dark at RT. *L*-methionine (5 mM final) was added to prevent methionine oxidation in tryptic peptides. Extraction of peptides from solution and SRM quantification were accomplished as mentioned above. Raw files for each sample were recorded and analyzed with Skyline software, and peptide areas were used to calculate light-to-heavy ratios and TEX101 concentration in each sample.

### Statistics

Power calculations were done with G*Power software (version 3.1.7, Heinrich Heine University, Dusseldorf, Germany). GraphPad Prism (v4.0; Graphpad Software, San Diego, CA, USA) was used to generate scatterplots, perform statistical analysis, and calculate the area under the Receiver Operating Characteristic curve (ROC AUC) and diagnostic sensitivity and specificity. Comparisons for two groups were made using the non-parametric Mann–Whitney U test, while multiple groups were analyzed by Kruskal–Wallis test, followed by Dunn’s multiple comparison test. Because only 30 of 64 pre-vasectomy samples were matched to post-vasectomy samples, unpaired Mann–Whitney U analysis was applied. All hypotheses testing was two-tailed, and *P-*values <0.05 were considered statistically significant. The assay’s reference interval was estimated using the pre-vasectomy SP samples. TEX101 values were log_10_-transformed and the lower and upper 95% confidence interval (CI) of the arithmetic mean was calculated. Correlations between TEX101 concentration and other continuous variables were assessed by Spearman correlation coefficients (r_s_). For the first and second intended uses (evaluation of vasectomy efficiency and differential diagnosis of azoospermia forms), the cut-offs and sensitivities were determined based on 100% specificity. For the third intended use (prediction of sperm retrieval), the cut-off, sensitivity, and specificity were exploratory. Samples with missing data were not used in calculations.

### Study approval

Semen samples were obtained with informed consent from patients by masturbation with 2–5 days of abstinence before collection. Eight hundred and fifty (*N* = 850) semen samples were obtained from healthy, fertile men and patients diagnosed with unexplained infertility, oligospermia, and azoospermia (Table 1). Sample collection was approved by the institutional review boards of Mount Sinai Hospital (approval #08-117-E) and University Health Network (#09-0830-AE). Samples were collected as a convenience series and analyzed retrospectively. The time difference between initial sperm count measurements in semen and TEX101 measurements in SP varied from several months to up to 5 years.

**Table 1:** Clinicopathological variables of the patient cohort

Clinical parameters	Number	Percentage
Number of subjects	850	100
Age – median (range), years	41 (19–63)	
Ethnic group		
African-Canadian	28	3.3
Asian	68	8.0
Caucasian	271	31.9
Hispanic	11	1.3
Indo-Canadian	11	1.3
Middle Eastern	27	3.2
Native-Canadian	11	1.3
Unspecified/unavailable	423	49.8
Diagnosis (sperm count in million/mL)		
Pre-vasectomy	67 (>15)	7.9
Post-vasectomy	63 (0)	7.4
Unexplained infertility	283 (15–360)	33.3
Oligospermia	276 (0.01–15)	32.5
Azoospermia	20 (0–0.5)	2.3
Non-obstructive azoospermia	100 (0–0.24)	11.8
Obstructive azoospermia	39 (0–0.18)	4.6
Unknown diagnosis	2	0.2

## Results

### Measurement of TEX101 in 821 seminal plasma samples using guanidine-based treatment protocol

We initially measured TEX101 in 821 SP samples obtained from healthy fertile men pre- and post-vasectomy, as well from patients with unexplained infertility, oligospermia, and OA and NOA. SP samples and ELISA standards were treated with 3 M GndCl at pH 12 for 1 h at RT, before analysis.

Results revealed high TEX101 concentrations in SP of fertile pre-vasectomy men (median 3433 ng/mL, *N* = 65), while it was undetectable in post-vasectomy men (median 0.5 ng/mL, *N* = 61). Similarly, TEX101 values were high in the group of men with unexplained infertility (median 2875 ng/mL, *N* = 276) and were significantly reduced in oligospermia (median 270.5 ng/mL, Mann–Whitney U test *P* < 0.0001, *N* = 269) and azoospermia (median 0.5 ng/mL, Mann–Whitney U test *P* < 0.0001, *N* = 150) samples (Additional file 2: Table S2 and Additional file 3: Figure S1). Because TEX101 is a germ cell-specific protein, we expected a strong correlation between its concentration in SP and the number of germ cells or spermatozoa in semen. The correlation between measured TEX101 concentration (ng/mL) and the sperm count (million/mL), however, was of moderate strength (r_s_ = 0.74). In addition, we noticed that a small fraction of patients (*N =* 17) with unexplained infertility (sperm count >15 million/mL) and oligospermia (sperm count >7 million/mL) had undetectable levels of TEX101 (<0.5 ng/mL) in SP (Additional file 4: Table S3). It should be mentioned that previous studies of *Tex101* ^*−/−*^ mouse knockout models revealed normal phenotypes, normal sperm morphology, and high sperm count, but absolute sterility of male mice [24]. Thus, we decided to examine in detail samples from these 17 patients with alternative methods, such as SRM mass spectrometry.

We thus measured TEX101 by SRM in SP and matched spermatozoa of the 17 patients with a high sperm count and undetectable TEX101 protein (Additional file 4: Table S3). TEX101 protein was detected in both SP and spermatozoa in all 17 patients. This allowed us to exclude the hypothesis of TEX101 gene knockouts in those patients. Additional examination of recent genomic data for loss-of-function mutations in *TEX101* supported our conclusions. According to the ExAC Browser (http://exac.broadinstitute.org), stop gain, frameshift, and splice donor mutations in *TEX101* were are and detected with the allele frequency <0.004% in a general population of 60,692 individuals.

### Isoform identity of TEX101 in spermatozoa and seminal plasma

**Fig. 2:**
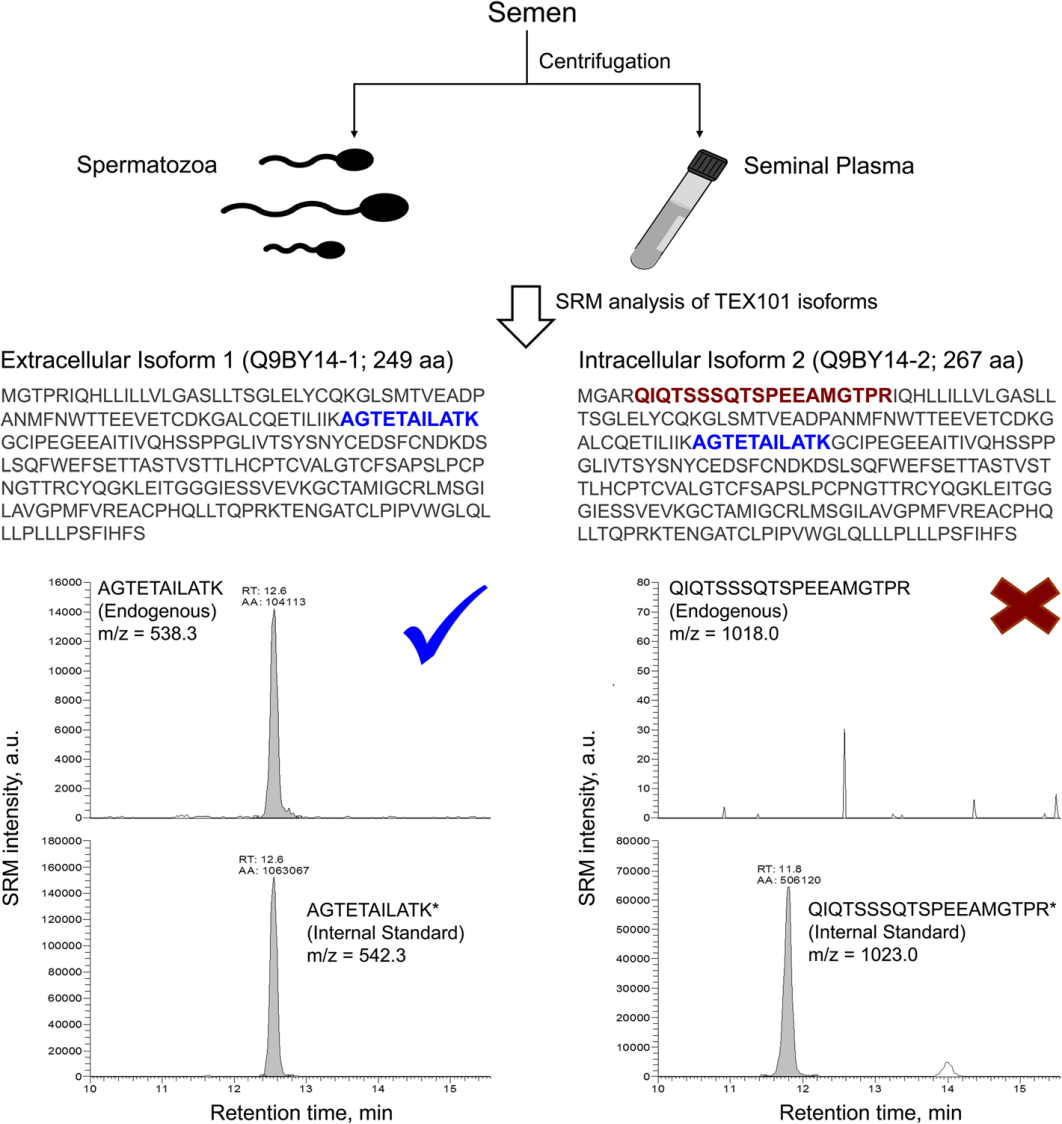
Investigation of TEX101 isoforms in spermatozoa and seminal plasma (SP) using selected reaction monitoring (*SRM*). Semen samples from men with a high sperm count (≥7 million/mL) and unexplained infertility and oligospermia (*N =* 17, see Additional file 4: Table S3) were centrifuged to separate spermatozoa from SP, which were then analyzed by SRM. Peptide AGTETAILATK common for both isoforms was used to quantify total TEX101, while unique peptide QIQTSSSQTSPEEAMGTPR was used to detect the presumed intracellular TEX101 isoform Q9BY14-2. Corresponding heavy isotope-labeled peptides were used as internal standards. Intracellular isoform Q9BY14-2 was not detected in either spermatozoa or SP, so total TEX101 was assumed to be a 249 amino acid (*aa*) extracellular membrane isoform Q9BY14-1. The checkmark indicates detection and X the absence of the peptides shown

According to UniProt (www.uniprot.org), alternative splicing of human *TEX101* results in two isoforms: an extracellular membrane-bound isoform Q9BY14-1 (249 amino acids) and an intracellular isoform Q9BY14-2 (267 amino acids). To further investigate the identity of TEX101 in SP, we developed an SRM assay for the peptide QIQTSSSQTSPEEAMGTPR that was unique for the intracellular isoform Q9BY14-2 (Additional file 5: Figure S2). We hypothesized that the intracellular isoform Q9BY14-2 could be exclusively expressed in those 17 samples, but was not captured by our monoclonal antibody (generated against the extracellular isoform Q9BY14-1) and measured by ELISA. SRM measurements in SP and spermatozoa in these 17 samples revealed the absence of intracellular isoform Q9BY14-2 and the exclusive presence of the extracellular membrane-bound isoform Q9BY14-1 (Fig. 2).

### Confirmation of TEX101 germ cell-specificity and extracellular membrane localization by immunohistochemistry

**Fig. 3:**
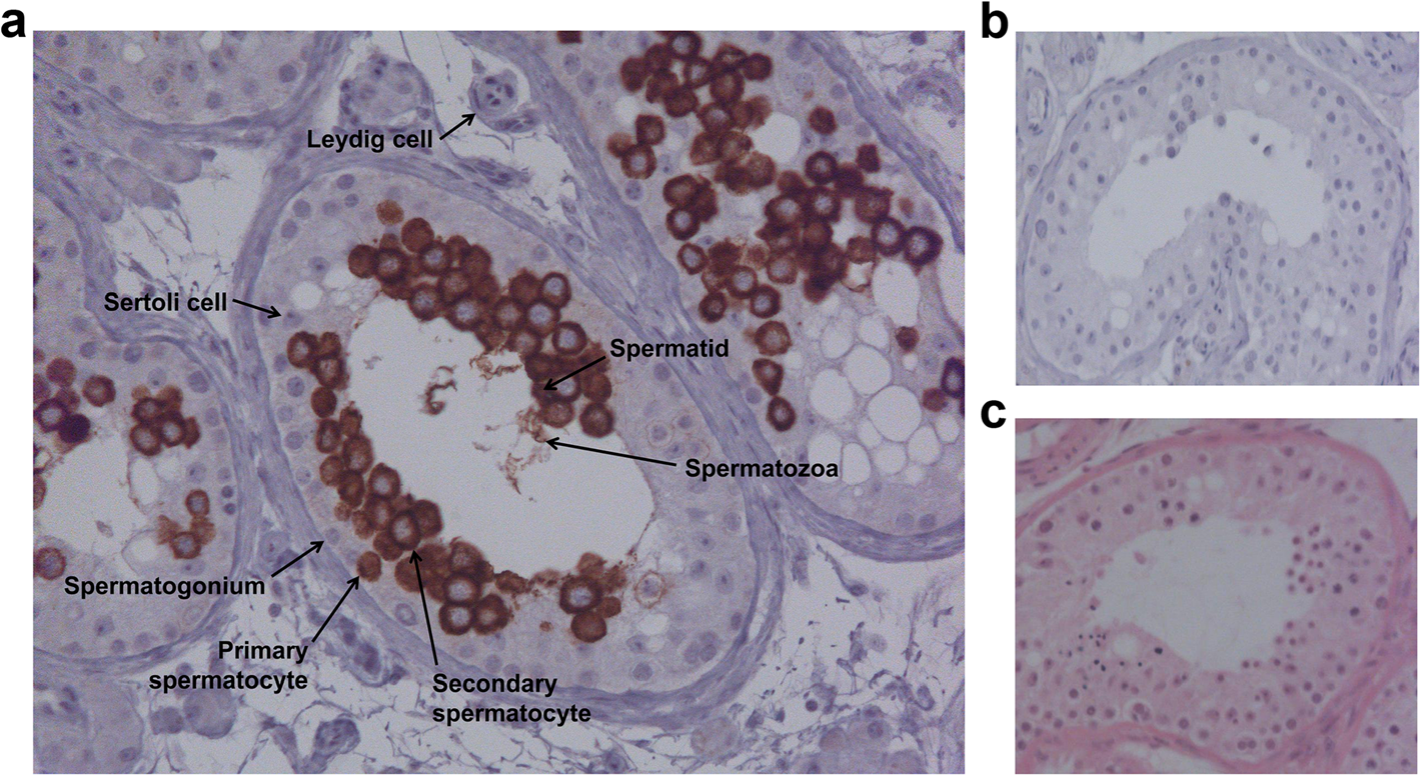
Immunohistochemical staining of TEX101 protein in testicular tissue with active spermatogenesis. **a** Testicular tissue stained with our monoclonal antibody 23ED228 (final concentration 80 ng/mL). Cell types presented include Sertoli cells (negative staining), Leydig cells (negative staining), and germ cells at different stages of spermatogenesis, such as spermatogonia (negative), primary spermatocytes (positive cytoplasm), secondary spermatocytes (positive membrane), spermatids (positive), and spermatozoa. **b** Negative control (no primary antibody added). **c** Hematoxylin and eosin staining of testicular tissue showing nucleus (*purple*) and cytoplasm (*pink*)

Previously, TEX101 protein expression in testicular tissues was studied using rabbit polyclonal antibodies generated against TEX101 peptide fragments (Atlas Antibodies HPA041915 and HPA042513). Here, we stained testicular tissues with normal spermatogenesis using our monoclonal antibody 23ED228, generated against the full TEX101 protein. We observed more intense staining of germ cells and lower background with our antibody at a final concentration of 80 ng/mL, versus 400 ng/mL for HPA041915 antibody. We observed no staining of Leydig, Sertoli, or spermatogonia cells; very weak cytoplasmic and membrane staining in primary spermatocytes; and very intense extracellular/membrane staining in secondary spermatocytes, spermatids, and testicular spermatozoa. Immunohistochemistry confirmed the exclusive expression of TEX101 in germ cells and its localization to the extracellular membrane (Fig. 3).

### Search for soluble TEX101 complexes in seminal plasma by size-exclusion chromatography

**Fig. 4:**
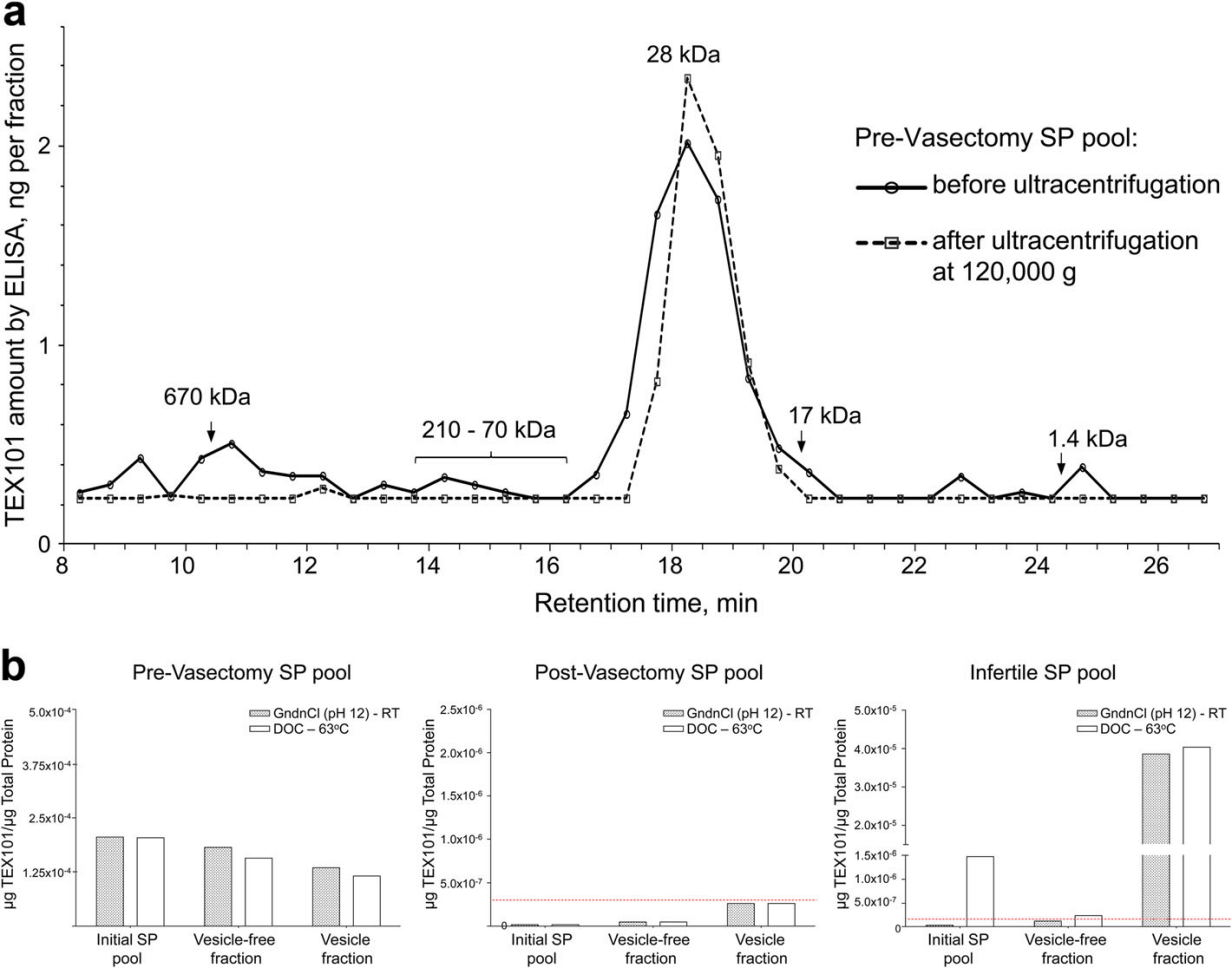
Investigation of TEX101 analyte identity in seminal plasma (*SP*) and the impact of SP pretreatment. **a** Size-exclusion chromatography was used to investigate TEX101 association with protein complexes and seminal microvesicles (SMVs) in the pre-vasectomy SP. Using molecular weight standards, we estimated the molecular weight of free soluble TEX101 as 28 kDa. No major complexes with other proteins were detected in the range 70–210 kDa. Some TEX101 (~14%), however, was associated with the high-molecular weight structures (>500 kDa) and found in the void volume. Ultracentifugation at 120,000 × *g* of the same pre-vasectomy SP pool followed by size-exclusion chromatography resulted in the non-detectable TEX101 in the high-molecular weight fractions. **b** The effect of guanidinium chloride (*GndCl*)- and sodium deoxycholate (*DOC*)-based treatments on TEX101 measurements by ELISA was estimated for SP pools of pre-vasectomy, post-vasectomy, or infertile men, as well as for their corresponding vesicle-free fractions and SMVs. *Dotted lines* represent the ELISA limit of detection. Both GndCl- and DOC-based treatments were efficient in the pre-vasectomy pools with high TEX101, with substantial amounts of TEX101 found associated with SMVs. GndCl-based treatment, however, was not efficient in the pool of infertility samples with low TEX101 (~100-fold lower than amounts in the pre-vasectomy pools

Previously, it was suggested that mouse TEX101 was involved in protein–protein interactions prior to its cleavage from the cell surface and release into SP [24, 25]. Because potential complexes of human TEX101 might hamper its capture or detection by antibodies with ELISA test, we used size-exclusion chromatography to investigate the presence of soluble TEX101 complexes in the pool of pre-vasectomy SP. Following DOC pretreatment at 63 °C of SP, ELISA was used to measure the level of TEX101 in each fraction. Results revealed that TEX101 was eluted as a single peak with 28 kDa size, as estimated by size-exclusion chromatography standards (Fig. 4a). This molecular weight was in good agreement with our previous estimates by western blot (~30 kDa for the glycosylated form and ~20 kDa for a deglycosylated form after PNGaseF treatment) [16].

We assumed that putative TEX101 complexes with other proteins would elute within fractions corresponding to higher molecular weights (i.e., >30 kDa). However, we detected negligible amounts of TEX101 (<8%) in those fractions (Fig. 4a, before ultracentrifugation) and concluded that the majority of TEX101 was present as an unbound free soluble form. We also noted that some TEX101 (~14% of total) was found in fractions corresponding to high-molecular weight molecules (>500 kDa) and the void volume. Ultracentifugation at 120,000 × *g* of the same pre-vasectomy SP pool followed by size-exclusion chromatography (Fig. 4a, after ultracentrifugation) resulted in non-detectable TEX101 in those high-molecular weight fractions. We thus hypothesized that a fraction of TEX101 might be associated with the debris of spermatozoa cellular membranes, exosomes, or SMVs.

### TEX101 association with seminal microvesicles in seminal plasma

All the above-mentioned experiments suggest that the failure of ELISA to measure low amounts of TEX101 in some SP samples (17 samples from unexplained infertility and oligospermia groups) was not related to TEX101 mutant forms, intracellular isoforms, or soluble complexes. To investigate if TEX101 was associated with SMVs, we pooled four of these 17 SP samples (two from each group) and isolated SMVs by centrifugation at 120,000 × *g*. As a control, we also isolated SMVs from pools of pre- or post-vasectomy SP samples. SMVs were denaturated with RapiGest SF at 65 °C, proteins were digested by trypsin, and TEX101 was quantified by the antibody-independent SRM assay in SMVs, matched vesicle-free supernatants, and original SP pools. Results confirmed the presence of TEX101 in SMVs and vesicle-free fractions of the four-sample infertility pool, as well as of the pre-vasectomy pool (Additional file 6: Figure S3). TEX101 levels in SMVs, matched vesicle-free supernatants, and initial SP of the post-vasectomy pool were below the limit of detection (LOD; 27 pg/μg of total protein).

We also measured by SRM in the pre-vasectomy SP and corresponding SMVs three highly tissue-specific secreted proteins that represent major glands in the male urogenital system. We detected high amounts of KLK3 (prostate-specific protein; 4.14 ng/μg of total protein), SEMG1 (seminal vesicle-specific protein; 37.3 ng/μg), ADAM7 (epididymis-specific protein; 0.45 ng/μg), and TEX101 (0.14 ng/μg) in the digest of SMVs. Interestingly, relative ratios of SEMG1, ADAM7, and TEX101 versus KLK3 were enriched 3.3-, 40-, and 12-fold in SMVs relative to the initial SP. No enrichment of these proteins (i.e., same ratios versus KLK3 in SMVs and SP) would indicate that SMVs non-specifically absorb KLK3, SEMG1, ADAM7, and TEX101. Substantial enrichment of ADAM7 and TEX101 in SMVs thus suggested the specific mechanism of their association with SMVs. Such a mechanism was previously demonstrated for ADAM7 protein [26]. Overall, the presence of prostate-, seminal vesicle-, testis-, and epididymis-specific proteins indicates that SMVs are produced by multiple glands in the male urogenital system, as previously discussed [27].

We then investigated which of two SP pretreatment protocols (GndCl- or DOC-based) would result in more complete SMV lysis and release of TEX101 and thus facilitate accurate measurement by ELISA. As a result, the same levels of TEX101 in the pre-vasectomy SP pool were measured with both treatment protocols in SMVs and vesicle-free SP (Fig. 4b). Substantial amounts of TEX101 were associated with vesicles, as previously shown by SRM. The level of TEX101 in the post-vasectomy SMVs and vesicle-free SP pool was below detection (<0.5 ng/mL) in all fractions (Fig. 4b).

Surprisingly, the pool of four infertility samples revealed the discrepancy between two pretreatment protocols. In infertile men, incubation of the original pool and the vesicle-free fraction with DOC at 63 °C identified a significantly higher level of TEX101 than pretreatment with GndCl at RT. GndCl treatment was thus less effective in the initial SP compared to the SMV fraction, suggesting some interaction between the SP matrix and SMVs (Fig. 4b). Based on these findings, we decided to re-examine the efficiency of treatments with GndCl at RT or DOC at 63 C and investigate any parameters that could affect the ELISA performance.

### Effect of seminal plasma pretreatment on TEX101 measurements by ELISA

Immunocapture-SRM was used to investigate the impact of SP pretreatment on the efficiency of TEX101 capture by the in-house-generated monoclonal 23ED616.8 and the commercial polyclonal ab69522 antibodies. Aliquots of pooled SP samples were mixed (1:1) with 6 M GndCl (pH 12) or 4% DOC and incubated at RT or 63 °C, respectively. Samples were analyzed in triplicate at 10-, 100-, and 1000-fold dilutions (Additional file 7: Figure S4).

Results confirmed our earlier observations that ab69522 captured the native form of TEX101 in SP. Incubation of SP with GndCl or DOC disrupted the capture of TEX101 by ab69522. As we showed previously [16], 23ED616.8 had a lower affinity for native TEX101 than ab69522. Sample pretreatment with detergents at 63 °C, however, resulted in more efficient capture, at least 10-fold higher than TEX101 capture after denaturation at 63 °C without detergents. We also noted that GndCl at 63 °C resulted in a slightly lower signal, possibly due to a higher impact on disruption of TEX101-antibody complexes.

Both GndCl- and DOC-based protocols worked equally well in SP with high amounts of TEX101 (~5000 ng/mL, pre-vasectomy samples). The GndCl-based protocol was compromised only in some infertility samples with low TEX101 levels (<300 ng/mL). Because detection of even very low TEX101 levels would indicate some residual spermatogenesis in testis and increased chances for sperm retrieval, it was critical to demonstrate the robust performance of the DOC-based protocol in all samples, including samples with very low amounts of TEX101 (~0.5 ng/mL). Considering all of the above, we re-measured our entire clinical cohort of SP samples by ELISA using the DOC-based protocol.

### Stability of TEX101 protein in semen

We previously demonstrated high stability of TEX101 protein in SP [16]. Because the use of our test may involve SP collection and storage at home, and then transportation of the whole semen prior to measurements in the clinical laboratory, we assessed TEX101 stability in whole semen stored at +4 °C for up to 14 days. A semen sample from a patient with unexplained infertility was obtained at the infertility clinic, allowed to liquefy at RT, and aliquoted. One aliquot was centrifuged immediately, and TEX101 was measured in the SP by ELISA using the DOC-based protocol. Four other aliquots of whole semen were stored at +4 °C for 5, 6, 9, and 14 days, centrifuged, and measured by TEX101 ELISA. As a result, TEX101 concentrations in SP slightly varied from day to day (2.4 ± 0.4 μg/mL, coefficient of variation = 16%), but no particular trends indicating TEX101 degradation were observed (Additional file 8: Figure S5). We thus concluded that TEX101 protein was stable in both SP and whole semen.

### Evaluation of TEX101 as a male infertility biomarker in a population of 805 men

**Fig. 5:**
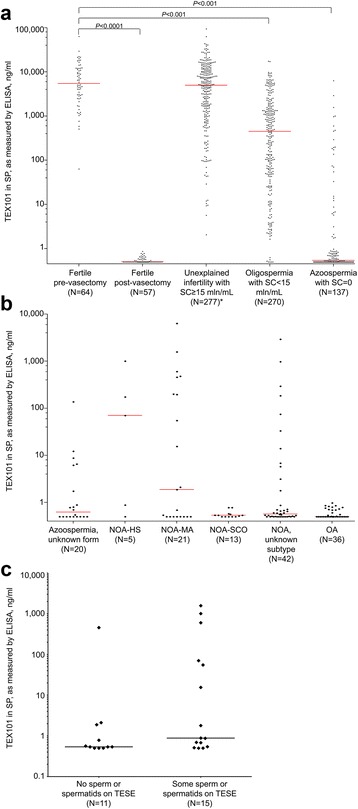
**a** TEX101 levels in seminal plasma (*SP*) of healthy, fertile pre- and post-vasectomy men and patients with unexplained infertility, oligospermia, and azoospermia, as measured by ELISA, using sodium deoxycholate (*DOC*)-based treatment. Median values for each group are presented as horizontal lines. TEX101 differentiated pre-vasectomy samples from post-vasectomy samples (Kruskal–Wallis test with the Dunn’s multiple comparison test *P*-value < 0.0001), oligospermia (*P* < 0.001), and azoospermia (*P* < 0.001), but not unexplained infertility (*P* > 0.05). **b** In the azoospermia group, TEX101 could differentiate between hypospermatogenesis (*HS*) and Sertoli cell-only syndrome (*SCO*) (Mann–Whitney U test *P* = 0.0336), but not between maturation arrest (*MA*) and SCO (*P* = 0.10). **c** Prediction of spermatozoa or spermatids retrieval in non-obstructive azoospermia (*NOA*) patients using TEX101 ELISA and DOC-based treatment of SP. *OA* obstructive azoospermia, *SC* sperm count, *TESE* testicular sperm extraction. *Female factor is not known for this group

TEX101 levels were measured in 805 SP samples using the DOC-based pretreatment protocol (Fig. 5a and Additional file 9: Table S4). The assay’s LOD was calculated as 0.5 ng/mL. TEX101 was detected in high amounts in all pre-vasectomy samples (median 5436 ng/mL, *N* = 64), but was undetectable in the post-vasectomy SP (*N* = 57). It should be noted that a fraction of post-vasectomy samples showed higher than usual background fluorescence (resulting in TEX101 levels between 0.5 and 0.9 ng/mL). Such elevated background, however, did not affect the proposed clinical cut-offs. TEX101 levels were high in the unexplained infertility group (median 4967 ng/mL, *N* = 277), and significantly lower in the oligospermic (median 450 ng/mL, *N* = 270) and azoospermic (median 0.54 ng/mL, *N* = 137) groups (Kruskal–Wallis test *P* < 0.0001). Likewise, differences between paired groups were also significant (Dunn’s multiple comparison test *P*-values <0.001), apart from the pre-vasectomy versus unexplained infertility group (*P* > 0.05) and post-vasectomy versus azoospermia (*P* > 0.05). Interestingly, comparison of 30 matched pre-vasectomy (median 7014 ng/mL) and post-vasectomy men (median 0.5 ng/mL) revealed that median TEX101 concentration decreased at least 13,500-fold after vasectomy, with the maximum decrease of 113,000 fold.

**Fig. 6:**
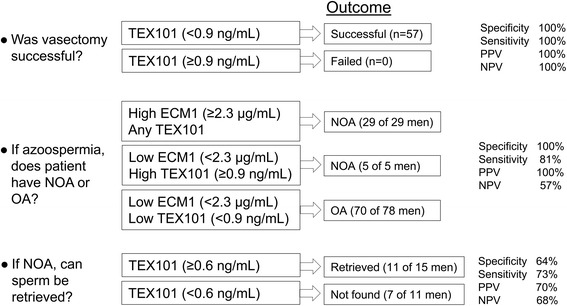
Clinical utility of TEX101 ELISA as a test to evaluate vasectomy success, differentiate between non-obstructive azoospermia (*NOA*) and obstructive azoospermia (*OA*), and predict the success of sperm retrieval in patients with NOA. A cut-off value of 0.9 ng/mL provided 100% sensitivity at 100% specificity, with an area under the Receiver Operating Characteristic curve (ROC AUC) of 1.00 (95% CI 1.00–1.00), for distinguishing pre- and post-vasectomy men. A combination of TEX101 ≥ 0.9 ng/mL with epididymis-specific protein ECM1 ≥ 2.3 μg/mL provided 81% sensitivity at 100% specificity to differentiate between NOA and OA. A TEX101 cut-off value of ≥0.6 ng/mL provided 73% sensitivity at 64% specificity and ROC AUC of 0.69 (95% CI 0.48–0.89) to predict sperm retrieval in patients with NOA. *NPV* negative predictive value, *PPV* positive predictive value

Based on 64 pre-vasectomy samples, the normal range of TEX101 in the healthy, fertile men was estimated between 515.1 ng/mL (95% CI 386.8–686.0) and 50,360 ng/mL (95% CI 37,817–67,065). Our data confirmed that TEX101 was a highly informative biomarker to predict the success of vasectomy or vasectomy reversal [11]. TEX101 at >0.9 ng/mL differentiated between pre- and post-vasectomy samples with 100% sensitivity at 100% specificity and ROC AUC 1.00 (95% CI 1.00–1.00). We thus suggest that TEX101 has a clinical utility to non-invasively evaluate the success of vasectomy or vasectomy reversal (Fig. 6).

TEX101 was not a useful marker of unexplained infertility (AUC = 0.56, 95% CI 0.48–0.63, *P* > 0.05), but performed well in oligospermia (AUC = 0.88, 95% CI 0.84–0.92, *P* < 0.001) and azoospermia (AUC = 0.99, 95% CI 0.98–1.00, *P* < 0.001). A cut-off value of 496 ng/mL provided 96% sensitivity at 98% specificity for distinguishing fertile pre-vasectomy men from patients with azoospermia.

TEX101 levels were broadly distributed in the azoospermia group with the unknown form (median 0.6 ng/mL), NOA with the unknown histological subtype (0.6 ng/mL), and in NOA-HS (70.4 ng/mL) and NOA-MA (1.9 ng/mL). Levels in the NOA-SCO subtype and OA were mainly below the LOD of 0.5 ng/mL (Fig. 5b). Based on our small set of NOA samples with the biopsy-confirmed histological subtypes, TEX101 could differentiate between HS and SCO (Mann–Whitney U test *P* = 0.0336), but not between MA and SCO (*P* = 0.10). Additional studies with a larger sample size are required to investigate if TEX101 can differentiate between histological subtypes of NOA.

Finally, the correlation between TEX101 levels and sperm count improved (r_s_ = 0.83, *P* < 0.001 with DOC-based protocol versus r_s_ = 0.74 with GndCl-based protocol), but was still not very strong (Additional file 10: Figure S6). This fact suggested that (i) TEX101 expression per germ cell may vary in different individuals; (ii) the fraction of TEX101 cleaved from the surface may vary; or (iii) TEX101 was released into SP not only by epididymal spermatozoa, but also by testicular germ cells.

### Evaluation of TEX101 as a biomarker to differentiate between non-obstructive azoospermia and obstructive azoospermia

TEX101 ≥ 0.9 ng/mL differentiated between NOA (*N =* 81) and OA/post-vasectomy (*N =* 93) with an AUC of 0.67 (95% CI 0.59–0.75, *P =* 0.00012) and 32% sensitivity at 99% specificity. Thus, TEX101 alone was not a strong marker for the non-invasive differentiation between NOA and OA. Previously, we proposed that a combination of epididymis-specific protein ECM1 (measured by ELISA) and TEX101 (measured by mass spectrometry) could differentiate between NOA and OA and thus eliminate the majority of diagnostic testicular biopsies [14].

Here, we tested the combination of ECM1 and TEX101, both measured by ELISA, in NOA (*N =* 42) and OA/post-vasectomy (*N =* 70) samples. TEX101 ≥ 0.9 ng/mL detected five additional NOA cases missed by ECM1 ≥ 2.3 μg/mL and thus increased sensitivity to detect NOA from 69% (ECM1 alone) to 81%, at 100% specificity. We have chosen such cut-offs to provide 100% specificity, at the expense of lower sensitivity. Assuming a 20% prevalence of OA in the azoospermic population, the combination of ECM1 and TEX101 provided 100% positive and 57% negative predictive values. All azoospermia cases diagnosed as NOA by ECM1 ≥ 2.3 μg/mL and TEX101 ≥ 0.9 ng/mL will thus be correct (32 patients), while 19% of patients diagnosed as OA based on ECM1 < 2.3 μg/mL and TEX101 < 0.9 ng/mL (eight patients in the current set) will be actually NOA (false negatives). We believe that false negatives are acceptable because presumed OA patients would be followed up with sperm retrieval, which would re-classify false OA as NOA. In combination with ECM1, non-invasive differentiation between NOA and OA is another clinical utility of TEX101 (Fig. 6). A combined ECM1 and TEX101 test may eliminate the majority of diagnostic testicular biopsies.

### Evaluation of TEX101 as a biomarker to predict sperm retrieval in non-obstructive azoospermia patients

Twenty-six NOA patients in our cohort underwent TESE, a surgical procedure to retrieve spermatozoa or spermatids from the testis for use in *in vitro* fertilization. Here, we evaluated the clinical utility of TEX101 to predict spermatozoa or spermatids retrieval by TESE in NOA patients. Note that useful biomarkers to predict sperm retrieval are currently not available. Testicular biopsy followed by histological subtyping thus remains the only practical diagnostic procedure.

Overall, the rate of successful sperm retrieval in NOA patients was previously estimated as 53% [28]. Rates varied for different histological subtypes of NOA: 81% for HS, 21% for MA, and 31% for SCO subtypes [28]. In our cohort of NOA samples with known histological subtypes and measured TEX101 (20 out of 26 samples in Fig. 5c), success rates for the corresponding subtypes were 100%, 55%, and 0%, respectively. Previously proposed biomarkers, such as follicle-stimulating hormone in blood, had moderate predictive efficiency (AUC = 0.72) with 70% sensitivity at 62% specificity [29].

Analysis of our clinical cohort (Fig. 5c) revealed TEX101 AUC = 0.69 (95% CI 0.48–0.89). With the cut-off of ≥0.6 ng/mL, TEX101 had 73% sensitivity, 64% specificity, 70% positive, and 68% negative predictive values (Fig. 6). Based on our small study with 26 patients, TEX101 alone had moderate diagnostic value as a predictor for spermatozoa or spermatid retrieval rate in patients with NOA. Owing to its germ cell-specificity, however, we suggest that TEX101 should be thoroughly evaluated in a larger cohort of NOA patients.

## Discussion

Immunoassays, such as sandwich ELISA, are indispensable tools for quantification of proteins in biological and clinical samples. The availability of ELISAs facilitates the clinical validation of putative protein biomarkers and enables their translation into diagnostic laboratory tests. The clear advantages of ELISAs over other methods for protein quantification include high analytical sensitivity, selectivity in complex biological matrices, high-throughput analysis, low reagent costs, simple execution, and straightforward interpretation of results [30].

Novel protein assays should be thoroughly evaluated prior to their use in the clinic. Clinical evidence of a novel diagnostic test includes scientific evidence (association of an analyte with the clinical condition), analytical performance (analytical sensitivity, selectivity, LOD, linearity, and reproducibility), and clinical performance (data to support reference ranges). It should be emphasized that different protein assay platforms, such as ELISA or mass spectrometry, may result in different reference values and different diagnostic performance for the same protein biomarker owing to different analyte identities (peptide or protein, free or bound form, linear or conformational epitope) and the use of different calibration standards [31].

Many novel diagnostic tests never make it to the clinic or even clinical trials. For instance, only 22 novel protein-based tests were approved by the Food and Drug Administration between 1993 and 2008 [32]. To improve the quality of diagnostic accuracy studies and facilitate clinical trials of only true biomarkers, the Standards for Reporting of Diagnostic Accuracy Studies (STARD) statement was proposed [17]. A recent update of STARD 2015 suggested reporting 30 essential items for the novel diagnostic assays. In this work, we followed STARD 2015 recommendations to present out novel TEX101 ELISA (Additional file 11: Table S5).

Our previous work on TEX101 as a biomarker of azoospermia [14] motivated us to generate monoclonal antibodies and develop a first-of-a-kind TEX101 ELISA [16]. It should be noted that SP is not a conventional fluid for clinical diagnostics and has some distinct differences, such as fast protease-mediated liquefaction, high viscosity, and an abundance of SMVs, which can hamper the performance of immunoassays. We realized that SP might need additional treatment procedures to facilitate protein quantification with high sensitivity. To improve ELISA sensitivity, we tested multiple combinations of detergents, temperatures, and pH values to select two SP pretreatment protocols: GndCl (pH 12) at RT and DOC at 63 °C [16]. Successful analysis of 10 pre- and 10 post-vasectomy samples and the scaled-up production of monoclonal antibodies encouraged us to design the large preclinical validation of TEX101 ELISA in a cohort of more than 800 SP samples available in our biobank.

We first implemented our assay with a GndCl-based protocol due to its simplicity and pretreatment at RT. Overall, the performance of the test was impressive, with absolute discrimination between healthy pre-vasectomy men and patients with OA or post-vasectomy. While reviewing the results, however, we noticed that 17 samples from the unexplained infertility and oligospermia groups with a substantial sperm count (≥7 million/mL) exhibited undetectable levels of TEX101 in SP (Additional file 3: Figure S1).

Comprehensive characterization of the analyte identity of our TEX101 ELISA was thus required. Mass spectrometry analysis of TEX101 in SP and spermatozoa suggested the exclusive presence of the extracellular membrane isoform Q9BY14-1 and the absence of intracellular isoform Q9BY14-2, which could potentially be undetectable by ELISA. Immunohistochemistry staining of testicular tissues confirmed the exclusive expression of TEX101 in germ cells and its extracellular membrane localization. Our initial hypothesis for mutant TEX101 protein in the infertile men was rejected by detection of TEX101 in both SP and corresponding spermatozoa by mass spectrometry. Size-exclusion chromatography confirmed the molecular weight of TEX101 (28 kDa) and did not reveal any additional TEX101-protein complexes in SP, but suggested an association of a small fraction of TEX101 with high-molecular weight structures.

Numerous studies previously suggested the presence of SMVs, such as prostasomes and epididymosomes, in semen [27, 33–35]. SMVs are produced by glands of the male urogenital tract and modulate spermatozoa maturation, transport, and capacitation. Even though TEX101 protein is not expressed in epididymis, it was previously identified in the epididymosome fractions [36]. However, it is still not clear if soluble TEX101 is simply absorbed by epididymis-secreted vesicles or is present in the vesicles produced in the lumen of seminiferous tubules (such as residual cytoplasm of spermatids) and co-purified with epididymosomes.

ELISA results revealed that both GndCl- and DOC-based protocols performed equally well in the pre-vasectomy pools including initial SP, vesicle-free SP, and the SMVs fraction. However, DOC-based treatment demonstrated superior efficiency in the infertility pool than GndCl-based treatment or denaturation by heat only. We thus believe that when TEX101 levels are very high (~5000 ng/mL in the pre-vasectomy pool), the impact of vesicles is negligible. However, when TEX101 levels are very low (<300 ng/mL in the infertility pool), the impact of SMVs becomes significant. Our results suggest that, unlike the DOC-based protocol, the GndCl-based protocol might not fully release TEX101 associated with SMVs in the matrix of SP. We would like to emphasize that these effects were noticeable only when TEX101 levels in SP were very low.

Finally, we re-measured our SP samples using the DOC-based pretreatment protocol. Note that the range of measured TEX101 concentrations in SP exceeded 127,000-fold (0.5–63,825 ng/mL). This is an unprecedented range of protein concentration in biological fluids, exceeding the 50,000-fold range for C-reactive protein [37] and 70,000-fold range for human chorionic gonadotropin in blood serum [38]. We defined the reference intervals of TEX101 in the healthy pre-vasectomy population and demonstrated clinical utility of TEX101 as a biomarker to predict the success of vasectomy or vasectomy reversal (Fig. 6). TEX101 differentiated between pre- and post-vasectomy samples with 100% sensitivity and 100% specificity, which is an extraordinary performance for a protein biomarker in biological fluids. In future, the TEX101 assay may be implemented as a home-based test and replace hospital-based sperm counting for patients after vasectomy or vasectomy reversal.

TEX101 alone was not a highly informative marker for non-invasive differentiation between NOA and OA (32% sensitivity at 99% specificity). However, the combination of TEX101 with ECM1 increased sensitivity to detect NOA from 69% (ECM1 alone) to 81%, both at 100% specificity. With our two-marker test, a fraction of NOA patients could be misdiagnosed as OA. Because TESE is recommended for all OA patients owing to the very high chances of sperm retrieval, all misdiagnosed patients will undergo TESE and therefore be re-classified. The decision to proceed with TESE is critical for NOA patients, for whom the chances of successful TESE are not known and may be quite low. Some OA patients misdiagnosed as NOA may choose to avoid TESE, despite being fertile through TESE and assisted reproduction techniques. Thus, it is acceptable to misdiagnose some NOA patients as OA, but not vice versa. We thus suggest that differentiation between NOA and OA with ECM1 and TEX101 is another clinical utility of TEX101. Such a test is non-invasive and will eliminate the majority of diagnostic testicular biopsies.

Our test also revealed that TEX101 could be an informative biomarker to predict sperm or spermatid retrieval (AUC = 0.69; 73% sensitivity at 64% specificity). It should be noted that the outcome of sperm retrieval by TESE, which is the clinical reference standard in this case, may depend on the length of surgery and be different in different clinics. It is still possible that some rare focal spermatogenesis could be present in some seminiferous tubules, but missed during surgery. TEX101 levels in SP may reflect the cumulative yield of spermatogenesis and thus be useful to detect rare focal spermatogenesis.

The prediction of sperm retrieval by TEX101 was comparable to other biomarkers that predict sperm retrieval, including seminal protein LGALS3BP (AUC = 0.76; 100% sensitivity at 45% specificity) [39], seminal leptin (AUC = 0.59; 43% sensitivity at 75% specificity) [40], seminal ESX1 mRNA (84% sensitivity at 28% specificity) [41], and blood serum FSH protein (AUC = 0.62; 71% sensitivity at 68% specificity) [40]. Better biomarkers to predict sperm retrieval in NOA patients are still required. Ultimately, the germ cell-specificity of TEX101 protein warrants its thorough validation in a large cohort of NOA patients.

## Conclusions

In this study we presented an optimized TEX101 immunoassay and its preclinical evaluation in a large set of SP samples. We propose to implement our TEX101 ELISA as a clinical test to evaluate vasectomy success, stratify azoospermia forms and subtypes, and predict the success of sperm retrieval in NOA patients. It should be noted that SP is a promising but unconventional fluid for clinical diagnostics. Our work revealed potential issues with SP as a fluid for clinical analyses, demonstrated solutions for the measurement of protein analytes in SP, and paved the road to translation of SP-based diagnostic tests into clinical practice.

## Additional files

Additional file 1: **Table S1.** Parameters of SRM assay of TEX101 protein. (PDF 10.3 kb)

Additional file 2: **Table S2.** Column statistics for TEX101 analysis in SP samples (*N* = 821) by ELISA using GndCl-based treatment protocol. (PDF 9.8 kb)

Additional file 3: **Figure S1.** TEX101 levels measured in SP samples (*N* = 821) by ELISA using GndCl-based protocol. (PDF 25.4 kb)

Additional file 4: **Table S3.** Diagnosis, sperm count, and TEX101 levels in SP of 17 infertile men with high sperm count. (PDF 75.7 kb)

Additional file 5: **Figure S2.** Quantification of total and intracellular TEX101 in pooled SP samples by SRM assays. (PDF 21.4 kb)

Additional file 6: **Figure S3.** Amount of TEX101 per microgram of digested total protein in different SP pools and their respective vesicle-free and vesicles fractions, as measured by SRM. (PDF 72.3 kb)

Additional file 7: **Figure S4.** Relative amounts of TEX101 captured from SP pools by commercial (mPoly) or in-house-generated (23-ED-616.8) antibodies, as measured by SRM. (PDF 22.2 kb)

Additional file 8: **Figure S5.** TEX101 stability in the whole semen. (PDF 135 kb)

Additional file 9: **Table S4.** Column statistics for TEX101 analysis in seminal plasma samples (*N* = 805) using DOC-based protocol. (PDF 9.7 kb)

Additional file 10: **Figure S6.** Correlation between sperm count and TEX101 levels in SP. (PDF 18.5 kb)

Additional file 11: **Table S5.** The list of Standards for Reporting of Diagnostic Accuracy Studies (STARD2015) recommendations followed in the present work. (PDF 16.4 kb)

## Abbreviations

AUC: Area under the curve
BCA: bicinchoninic acid
BSA: Bovine serum albumin
CI: Confidence interval
DOC: Sodium deoxycholate
DTT: dithiothreitol
ELISA: Enzyme-linked immunosorbent assay
GndCl: Guanidinium chloride
HS: Hypospermatogenesis
LOD: Limit of detection
MA: Maturation arrest
NOA: Non-obstructive azoospermia
OA: Obstructive azoospermia
PBS: phosphate-buffered saline
ROC: Receiver Operating Characteristic curve
RT: Room temperature
SCO: Sertoli cell-only syndrome
SMVs: Seminal microvesicles
SP: Seminal plasma
SRM: Selected reaction monitoring
STARD: Standards for Reporting of Diagnostic Accuracy Studies Statement
TESE: testicular sperm extraction
