# Supplementary material for: Preclinical evaluation of a TEX101 protein ELISA test for the differential diagnosis of male infertility
Source: BMC Med. 2017 Mar 23;15:60. doi: 10.1186/s12916-017-0817-5 (PMC5363040; doi:10.1186/s12916-017-0817-5)
Supplement: Supplementary file 1 — Table S1. Parameters of SRM assay of TEX101 protein. (PDF 10.3 kb) [file 12916_2017_817_MOESM1_ESM.pdf]

Additional file 1: Table S1. Parameters of SRM assay of TEX101 protein. K*, heavy isotope-labeled *L*-Lysine ($^{13}C_6$, $^{15}N_2$); R*, heavy isotope-labeled *L*-Arginine ($^{13}C_6$, $^{15}N_4$); IS, spike-in internal standard; Q1 and Q3; first and third quadrupoles of the triple quadrupole mass spectrometer; CE, collision energy. Heavy isotope-labeled peptides AGTETAILATK and QIQTSSSQTSPEEAMGTPR included C-term quantifying JPT tags (serine-alanine-[3-nitro] tyrosine-glycine) which were cleaved by trypsin prior to SRM analysis

TEX101 peptide	Q1 (m/z)	Q3 (m/z)	CE (V)	Scan time (ms)	Retention time (min)	Peptide type
**AGTETAILATK**	538.3	717.5	21.6	40	12.4	Light endogenous
**AGTETAILATK**	538.3	846.5	21.6	40	12.4	Light endogenous
**AGTETAILATK**	538.3	1004.6	21.6	40	12.4	Light endogenous
**AGTETAILATK***	542.3	725.5	21.6	40	12.4	Heavy IS
**AGTETAILATK***	542.3	854.5	21.6	40	12.4	Heavy IS
**AGTETAILATK***	542.3	1012.6	21.6	40	12.4	Heavy IS
**QIQTSSSQTSPEEAMGTPR**	1018.0	987.5	37.9	40	11.7	Light endogenous
**QIQTSSSQTSPEEAMGTPR**	1018.0	1390.6	37.9	40	11.7	Light endogenous
**QIQTSSSQTSPEEAMGTPR**	1018.0	1477.7	37.9	40	11.7	Light endogenous
**QIQTSSSQTSPEEAMGTPR***	1023.0	997.5	37.9	40	11.7	Heavy IS
**QIQTSSSQTSPEEAMGTPR***	1023.0	1400.6	37.9	40	11.7	Heavy IS
**QIQTSSSQTSPEEAMGTPR***	1023.0	1487.7	37.9	40	11.7	Heavy IS
**LMSGILAVGPMFVR**	745.9	649.3	28.7	40	26	Light endogenous
**LMSGILAVGPMFVR**	745.9	989.6	28.7	40	26	Light endogenous
**LMSGILAVGPMFVR**	745.9	1246.7	28.7	40	26	Light endogenous
