# Supplementary material for: Preclinical evaluation of a TEX101 protein ELISA test for the differential diagnosis of male infertility
Source: BMC Med. 2017 Mar 23;15:60. doi: 10.1186/s12916-017-0817-5 (PMC5363040; doi:10.1186/s12916-017-0817-5)
Supplement: Supplementary file 2 — Table S2. Column statistics for TEX101 analysis in SP samples (N = 821) by ELISA using GndCl-based treatment protocol. (PDF 9.8 kb) [file 12916_2017_817_MOESM2_ESM.pdf]

Additional file 2: Table S2. Column statistics for TEX101 analysis in SP samples (*N*=821) by ELISA using GndCl-based treatment protocol (3 M guanidine hydrochloride for 1 hour at RT). Q1: 25th percentile, Q3: 75th percentile, IQR: Interquartile range

**Samples**	**TEX101 (ng/mL)**				
	N	**Q1**	**Median**	**Q3**	**IQR**
Pre-Vasectomy	65	1,435	3,433	6938	5,504
Post-Vasectomy	61	0.5	0.5	0.5	0
Unexplained infertility	276	1,173	2,875	5,237	4,064
Oligospermia	269	21.8	271	972	950
Azoospermia	150	0.5	0.5	0.7	0.2
Azoospermia, unknown form	22	0.5	0.5	2.3	1.8
Non-obstructive azoospermia (NOA)					
Hypospermatogenesis	8	0.7	4	94.5	93.8
Maturation Arrest	22	0.5	0.9	175	174
Sertoli-cell only	13	0.5	0.5	0.5	0
Unknown histological subtype	51	0.5	0.5	0.8	0.3
Obstructive azoospermia (OA)	34	0.5	0.5	0.5	0
**TOTAL**	**821**	**0.8**	**429**	**2,654**	**2,654**
