# Supplementary material for: Preclinical evaluation of a TEX101 protein ELISA test for the differential diagnosis of male infertility
Source: BMC Med. 2017 Mar 23;15:60. doi: 10.1186/s12916-017-0817-5 (PMC5363040; doi:10.1186/s12916-017-0817-5)

Additional file 3: Figure S1. TEX101 levels measured in SP samples (*N*=821) by ELISA using GndCl-based protocol (3 M guanidine hydrochloride for 1 hour at RT). Median values for each group are shown as red bars. **Pre-V:** pre-vasectomy samples; **Post-V:** post-vasectomy samples; **Unexplained infertility:** infertile men with sperm count >15 mln/mL; **Oligospermia:** infertile men with sperm count <15 mln/mL; **Azoospermia:** men diagnosed with azoospermia (undetectable sperm in semen).

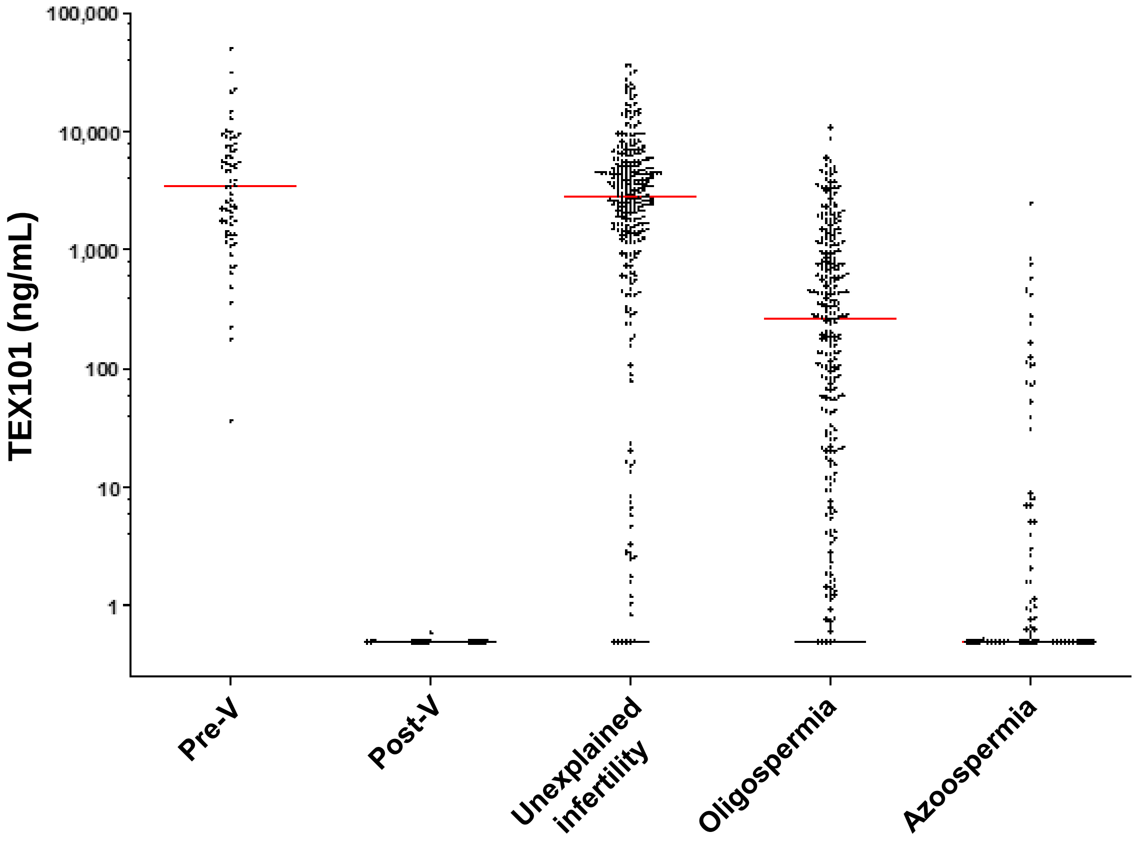

Supplement: Supplementary file 3 — Figure S1. TEX101 levels measured in SP samples (N = 821) by ELISA using GndCl-based protocol. (PDF 25.4 kb) [file 12916_2017_817_MOESM3_ESM.pdf]
