# Supplementary material for: Preclinical evaluation of a TEX101 protein ELISA test for the differential diagnosis of male infertility
Source: BMC Med. 2017 Mar 23;15:60. doi: 10.1186/s12916-017-0817-5 (PMC5363040; doi:10.1186/s12916-017-0817-5)
Supplement: Supplementary file 4 — Table S3. Diagnosis, sperm count, and TEX101 levels in SP of 17 infertile men with high sperm count. (PDF 75.7 kb) [file 12916_2017_817_MOESM4_ESM.pdf]

Additional file 4: Table S3. Diagnosis, sperm count and TEX101 levels in SP of 17 infertile men with high sperm count (≥7 mln/mL). Prior to ELISA measurements, samples were incubated with 3 M guanidine hydrochloride for 1 hour at RT. Notes: *****TEX101 protein was detected by additional peptide LMSGILAVGPMFVR, however accurate ratios based on internal standard could not be calculated due to its low levels (<LOQ) in these specific samples

Sample #	Group	Sperm count (mln/mL)	TEX101 in SP by ELISA (ng/mL)	TEX101 in SP by SRM (ng/mL)	TEX101 in spermatozoa by SRM (ng/μg total protein)
1	Unexplained infertility	111.1	<0.5	3051.8	0.11
2	Unexplained infertility	91.1	2.82	742.4	0.16
3	Unexplained infertility	30	<0.5	420.3	0.02
4	Unexplained infertility	58.8	<0.5	978.1	0.22
5	Unexplained infertility	18.5	<0.5	1140.3	0.08
6	Unexplained infertility	26.1	<0.5	Not Detected	0.13
7	Unexplained infertility	59	<0.5	2082.2	0.09
8	Unexplained infertility	104.5	<0.5	1099.7	0.05
9	Unexplained infertility	123.9	<0.5	389.3	0.15
10	Unexplained infertility	139.5	<0.5	1289.6	0.81
11	Oligospermia	6.9	<0.5	Not Detected	Detected*****
12	Oligospermia	10.3	<0.5	104.5	0.05
13	Oligospermia	7.7	<0.5	758.4	0.18
14	Oligospermia	8.4	<0.5	265.6	0.06
15	Oligospermia	9.9	<0.5	Not Detected	0.01
16	Oligospermia	10.9	<0.5	324.3	0.07
17	Oligospermia	12.8	<0.5	Detected*****	0.05
