# Supplementary material for: Preclinical evaluation of a TEX101 protein ELISA test for the differential diagnosis of male infertility
Source: BMC Med. 2017 Mar 23;15:60. doi: 10.1186/s12916-017-0817-5 (PMC5363040; doi:10.1186/s12916-017-0817-5)

Additional file 5: Figure S2. Quantification of total and intracellular TEX101 in pooled SP samples by SRM assays. The heavy isotope-labeled peptides AGTETAILATK* (total TEX101) and QIQTSSSQTSPEEAMGTPR* (intracellular TEX101) with a trypsin-cleavable JPT tag (serine-alanine-[3-nitro]tyrosine-glycine) were used as internal standards for the absolute quantification of endogenous TEX101 proteoforms. Calibration curves were generated by spiking increasing amounts of the internal standards into 1 µL of SP before proteomic sample preparation and trypsin digestion.

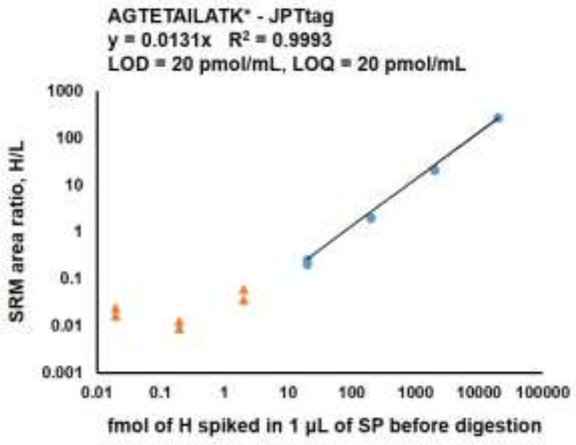

Supplement: Supplementary file 5 — Figure S2. Quantification of total and intracellular TEX101 in pooled SP samples by SRM assays. (PDF 21.4 kb) [file 12916_2017_817_MOESM5_ESM.pdf]
