# Supplementary material for: Preclinical evaluation of a TEX101 protein ELISA test for the differential diagnosis of male infertility
Source: BMC Med. 2017 Mar 23;15:60. doi: 10.1186/s12916-017-0817-5 (PMC5363040; doi:10.1186/s12916-017-0817-5)

Additional file 6: Figure S3. Amount of TEX101 per µg of digested total protein in different SP pools and their respective vesicle-free and vesicles fractions, as measured by SRM. Dotted red line represents LOD of SRM assay (27 pg/µg of total protein).

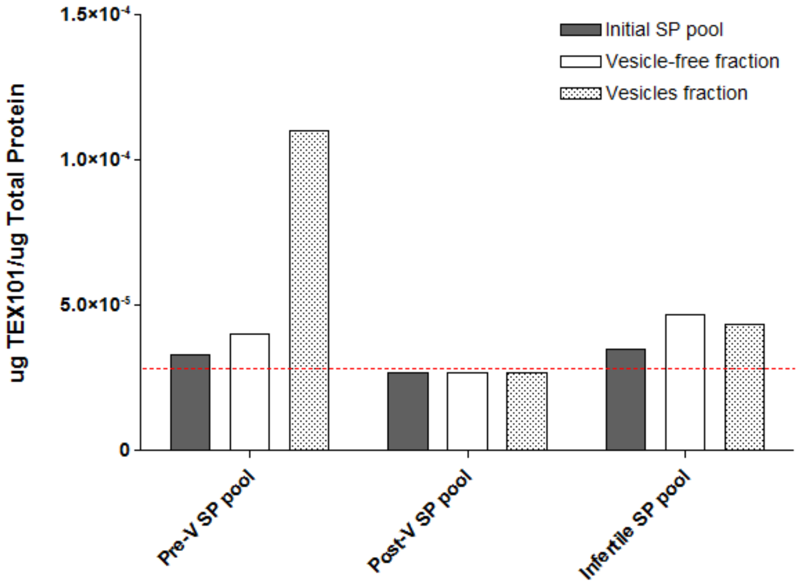

Supplement: Supplementary file 6 — Figure S3. Amount of TEX101 per microgram of digested total protein in different SP pools and their respective vesicle-free and vesicles fractions, as measured by SRM. (PDF 72.3 kb) [file 12916_2017_817_MOESM6_ESM.pdf]
