# Supplementary material for: Preclinical evaluation of a TEX101 protein ELISA test for the differential diagnosis of male infertility
Source: BMC Med. 2017 Mar 23;15:60. doi: 10.1186/s12916-017-0817-5 (PMC5363040; doi:10.1186/s12916-017-0817-5)

Additional file 7: Figure S4. Relative amounts of TEX101 captured from SP pools by commercial (mPoly) or in-house generated (23-ED-616.8) antibodies, as measured by SRM. SP samples were treated before capture using the following protocols: (1) untreated; (2) incubated with 3 M guanidine hydrochloride for 1 hour at RT; (3) incubated with 2% sodium deoxycholate for 1 hour at RT; (4) incubated with 3 M guanidine hydrochloride for 1 hour at 63°C; (5) incubated with 2% sodium deoxycholate for 1 hour at 63°C; and (6) incubated for 1 hour at 63°C.

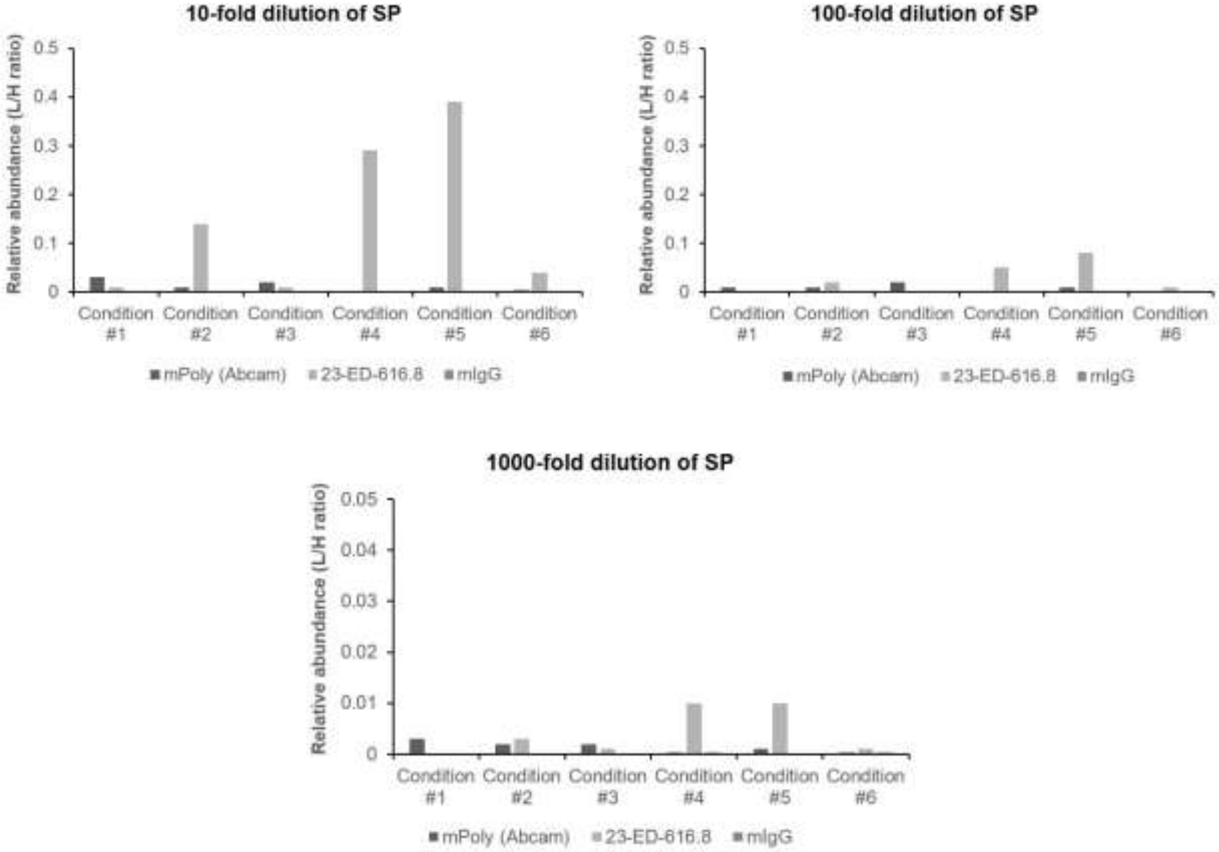

Supplement: Supplementary file 7 — Figure S4. Relative amounts of TEX101 captured from SP pools by commercial (mPoly) or in-house-generated (23-ED-616.8) antibodies, as measured by SRM. (PDF 22.2 kb) [file 12916_2017_817_MOESM7_ESM.pdf]
