# Supplementary material for: Preclinical evaluation of a TEX101 protein ELISA test for the differential diagnosis of male infertility
Source: BMC Med. 2017 Mar 23;15:60. doi: 10.1186/s12916-017-0817-5 (PMC5363040; doi:10.1186/s12916-017-0817-5)

Additional file 8: Figure S5. TEX101 stability in the whole semen. Semen samples were stored at +4Cº, centrifuged at each time point, and TEX101 was measured by ELISA with DOC-based protocol.

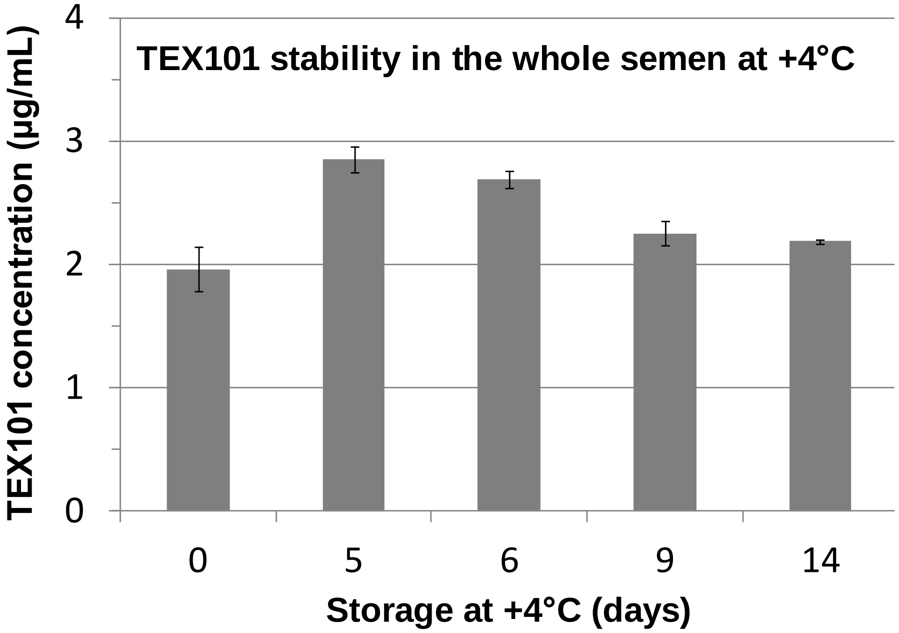

Supplement: Supplementary file 8 — Figure S5. TEX101 stability in the whole semen. (PDF 135 kb) [file 12916_2017_817_MOESM8_ESM.pdf]
