# Supplementary material for: Preclinical evaluation of a TEX101 protein ELISA test for the differential diagnosis of male infertility
Source: BMC Med. 2017 Mar 23;15:60. doi: 10.1186/s12916-017-0817-5 (PMC5363040; doi:10.1186/s12916-017-0817-5)
Supplement: Supplementary file 9 — Table S4. Column statistics for TEX101 analysis in seminal plasma samples (N = 805) using DOC-based protocol. (PDF 9.7 kb) [file 12916_2017_817_MOESM9_ESM.pdf]

Additional file 9: Table S4. Column statistics for TEX101 analysis in seminal plasma samples (*N*=805) using DOC-based protocol (2% sodium deoxycholate for 1 hour at 63°C). Q1: 25th percentile, Q3: 75th percentile, IQR: Interquartile range

**Samples**	**TEX101 (ng/mL)**				
	N	**Q1**	**Median**	**Q3**	**IQR**
Pre-Vasectomy	64	2,728	5,436	12,036	9,308
Post-Vasectomy	57	0.5	0.5	0.6	0.1
Unexplained infertility	277	1,325	4,967	11,013	9,688
Oligospermia	270	51	450	1,494	1,443
Azoospermia	137	0.5	0.5	0.9	0.4
Azoospermia, unknown form	20	0.5	0.6	4.0	3.5
Non-obstructive azoospermia (NOA)					
Hypospermatogenesis	5	0.7	70.4	590	590
Maturation arrest	21	0.5	1.9	329	328
Sertoli-cell only	13	0.5	0.5	0.6	0.1
Unknown histological subtype	42	0.5	0.6	2.5	2.0
Obstructive azoospermia (OA)	36	0.5	0.5	0.7	0.2
**TOTAL**	**805**	**5.8**	**771**	**5,016**	**5,010**
