# Supplementary material for: Preclinical evaluation of a TEX101 protein ELISA test for the differential diagnosis of male infertility
Source: BMC Med. 2017 Mar 23;15:60. doi: 10.1186/s12916-017-0817-5 (PMC5363040; doi:10.1186/s12916-017-0817-5)

Additional file 10: Figure S6. Correlation between sperm count and TEX101 levels in SP. Samples were incubated with 2% sodium deoxycholate for 1 hour at 63°C, prior to TEX101 analysis by ELISA. r_s, Spearman correlation coefficient.

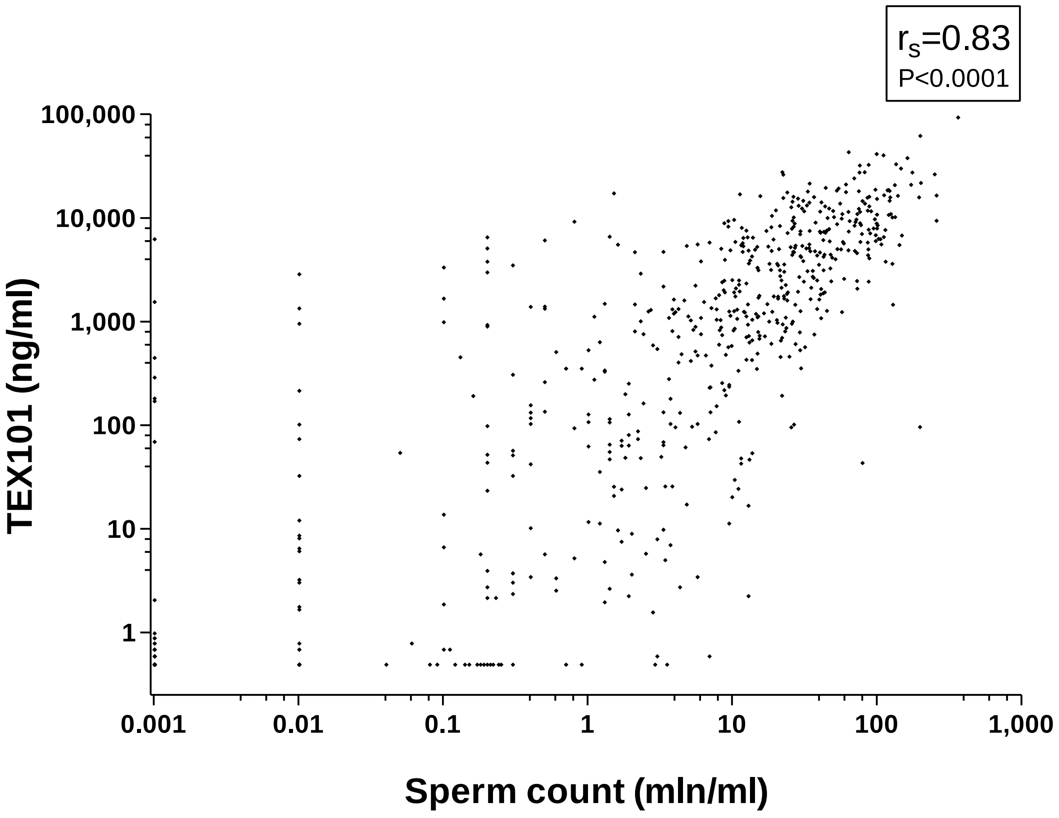

Supplement: Supplementary file 10 — Figure S6. Correlation between sperm count and TEX101 levels in SP. (PDF 18.5 kb) [file 12916_2017_817_MOESM10_ESM.pdf]
