# Supplementary material for: Preclinical evaluation of a TEX101 protein ELISA test for the differential diagnosis of male infertility
Source: BMC Med. 2017 Mar 23;15:60. doi: 10.1186/s12916-017-0817-5 (PMC5363040; doi:10.1186/s12916-017-0817-5)
Supplement: Supplementary file 11 — Table S5. The list of Standards for Reporting of Diagnostic Accuracy Studies (STARD2015) recommendations followed in the present work. (PDF 16.4 kb) [file 12916_2017_817_MOESM11_ESM.pdf]

Additional file 11: Table S5. The list of Standards for Reporting of Diagnostic Accuracy Studies (STARD2015) recommendations followed in the present work.

Section & Topic	No	Item	Reported on page #
**TITLE OR ABSTRACT**	**1**	Identification as a study of diagnostic accuracy using at least one measure of accuracy (such as sensitivity, specificity, predictive values, or AUC)	p. 1, 3
**ABSTRACT**	**2**	Structured summary of study design, methods, results, and conclusions (for specific guidance, see STARD for Abstracts)	p. 1
**INTRODUCTION**	**3**	Scientific and clinical background, including the intended use and clinical role of the index test	p. 2
	**4**	Study objectives and hypotheses	p. 2
**METHODS**			
Study design	**5**	Whether data collection was planned before the index test and reference standard were performed (prospective study) or after (retrospective study)	Introduction, p. 1<br>Methods, p. 2, 6
Participants	**6**	Eligibility criteria	Methods, p. 2
	**7**	On what basis potentially eligible participants were identified (such as symptoms, results from previous tests, inclusion in registry)	Methods, p. 2, 6
	**8**	Where and when potentially eligible participants were identified (setting, location and dates)	Methods, p.6
	**9**	Whether participants formed a consecutive, random or convenience series	Methods, p. 6
Test methods	**10a**	Index test, in sufficient detail to allow replication	Methods, p. 3, 4
	**10b**	Reference standard, in sufficient detail to allow replication	Methods, p. 3
	**11**	Rationale for choosing the reference standard (if alternatives exist)	Methods, p. 3
	**12a**	Definition of and rationale for test positivity cut-offs or result categories of the index test, distinguishing pre-specified from exploratory	Methods, p. 3, 5, 6
	**12b**	Definition of and rationale for test positivity cut-offs or result categories of the reference standard, distinguishing pre-specified from exploratory	Methods, p. 3
	**13a**	Whether clinical information and reference standard results were available to the performers/readers of the index test	Methods, p. 3
	**13b**	Whether clinical information and index test results were available<br>to the assessors of the reference standard	Methods, p. 3
Analysis	**14**	Methods for estimating or comparing measures of diagnostic accuracy	Methods, p. 5, 6
	**15**	How indeterminate index test or reference standard results were handled	NA
	**16**	How missing data on the index test and reference standard	NA

		were handled	
	**17**	Any analyses of variability in diagnostic accuracy, distinguishing pre-specified from exploratory	NA
	**18**	Intended sample size and how it was determined	Methods, p. 2, 6
**RESULTS**			
Participants	**19**	Flow of participants, using a diagram	Figure 1
	**20**	Baseline demographic and clinical characteristics of participants	Table 1
	**21a**	Distribution of severity of disease in those with the target condition	Table 1
	**21b**	Distribution of alternative diagnoses in those without the target condition	NA
	**22**	Time interval and any clinical interventions between index test and reference standard	Methods, p. 2, 6
Test results	**23**	Cross tabulation of the index test results (or their distribution) by the results of the reference standard	Figure 4c
	**24**	Estimates of diagnostic accuracy and their precision (such as 95% confidence intervals)	Methods, p. 5-6
	**25**	Any adverse events from performing the index test or the reference standard	NA
**DISCUSSION**	**26**	Study limitations, including sources of potential bias, statistical uncertainty, and generalisability	Discussion, p.13,14
	**27**	Implications for practice, including the intended use and clinical role of the index test	Discussion, p.14
**OTHER INFORMATION**	**28**	Registration number and name of registry	NA
	**29**	Where the full study protocol can be accessed	NA
	**30**	Sources of funding and other support; role of funders	Abstract, p.1
